# CCL2 signaling promotes skeletal muscle wasting in non-tumor and breast tumor models

**DOI:** 10.1242/dmm.050398

**Published:** 2024-09-09

**Authors:** Nadia Alissa, Wei Bin Fang, Marcela Medrano, Nick Bergeron, Yuuka Kozai, Qingting Hu, Chloe Redding, John Thyfault, Jill Hamilton-Reeves, Cory Berkland, Nikki Cheng

**Affiliations:** ^1^ Department of Cancer Biology, University of Kansas Medical Center, Kansas City, KS 66160, USA; ^2^ Department of Pathology and Laboratory Medicine, University of Kansas Medical Center, Kansas City, KS 66160, USA; ^3^ Department of Cell Biology and Physiology and Internal Medicine-Division of Endocrinology, University of Kansas Medical Center, Kansas City, KS 66160, USA; ^4^ Department of Urology, University of Kansas Medical Center, Kansas City, KS 66160, USA; ^5^ Department of Pharmaceutical Chemistry, University of Kansas, Lawrence, KS 66045, USA; ^6^ University of Kansas Cancer Center, Kansas City, KS 66160, USA

**Keywords:** Breast cancer, Cachexia, Skeletal muscle wasting, Chemokine, CCL2

## Abstract

Despite advancements in treatment, approximately 25% of patients with breast cancer experience long-term skeletal muscle wasting (SMW), which limits mobility, reduces drug tolerance and adversely impacts survival. By understanding the underlying molecular mechanisms of SMW, we may be able to develop new strategies to alleviate this condition and improve the lives of patients with breast cancer. Chemokines are small soluble factors that regulate homing of immune cells to tissues during inflammation. In breast cancers, overexpression of C-C chemokine ligand 2 (CCL2) correlates with unfavorable prognosis. Elevated levels of CCL2 in peripheral blood indicate possible systemic effects of this chemokine in patients with breast cancer. Here, we investigated the role of CCL2 signaling on SMW in tumor and non-tumor contexts. *In vitro*, increasing concentrations of CCL2 inhibited myoblast and myotube function through C-C chemokine receptor 2 (CCR2)-dependent mechanisms involving JNK, SMAD3 and AMPK signaling. In healthy mice, delivery of recombinant CCL2 protein promoted SMW in a dose-dependent manner. *In vivo* knockdown of breast tumor-derived CCL2 partially protected against SMW. Overall, chronic, upregulated CCL2–CCR2 signaling positively regulates SMW, with implications for therapeutic targeting.

## Teaser

**Editor's choice:** Using *in vitro* and *in vivo* models, this study identifies an important role for CCL2–CCR2 signaling in skeletal muscle wasting associated with breast cancer, with important implications for therapeutic targeting.

## INTRODUCTION

Breast cancer is the most commonly diagnosed form of cancer in women in the USA, with over 260,00 cases diagnosed every year (Giaquinto et al., 2022). Despite advancements in treatment, approximately 25% of patients with breast cancer experience long-term skeletal muscle wasting (SMW) (Mallard et al., 2021). SMW is a primary characteristic of cancer cachexia, a systemic wasting illness characterized by unexpected loss of body weight by ≥5% or body mass index by ≤20, along with anorexia, diminished strength and higher inflammation. SMW is associated with metastatic disease and is characterized by decreased muscle mass and strength. This contributes to increased fatigue, decreased mobility, and increased levels of anxiety and depression. Under severe circumstances, SMW impairs drug tolerance and respiration. As such, SMW has a significantly negative impact on patients with breast cancer by reducing the quality of life and overall survival (Amitani et al., 2022; Morishita et al., 2022; Simonavice et al., 2015; Sun et al., 2023). There is no cure for SMW. Skeletal muscle cannot be fully recovered in patients by increasing nutrition or exercise. As such, many patients with cancer are left with significant gaps in care (Biswas and Acharrya, 2020). Although SMW is considered more prevalent in patients with pancreatic, gastric or lung cancers, this condition may be underreported in patients with breast cancer (Biswas and Acharyya, 2020; Consul et al., 2016). By understanding the underlying molecular mechanisms of SMW, we may be able to develop new strategies to alleviate this condition and improve the lives of many patients with breast cancer.

Exercise and nutritional intake influence adult skeletal muscle mass (Janani and Sedhunivas, 2024; Martin-Cantero et al., 2021; Rosa-Caldwell et al., 2023), modulating cellular regeneration and breakdown. Reduced proliferation and differentiation of precursor myoblast cells prevents regeneration of skeletal muscle tissue. Loss of functioning muscle fibers occurs with breakdown of intracellular proteins in differentiated muscle cells. Skeletal muscle protein breakdown occurs through the ATP-dependent ubiquitin 26S proteasome, in which ubiquitin-labeled proteins are targeted and degraded. In this pathway, muscle proteins bind to atrogin-1 (also known as MAFbx or FBXO32) and muscle-specific RING finger protein 1 (MuRF1, also known as TRIM63), both muscle-specific E3 ligases. Activated ubiquitin is then transferred to the muscle proteins, targeting them for degradation (Martin et al., 2023; Sartori et al., 2021). Another mechanism of protein breakdown is cellular autophagy, in which organelles and proteins are removed by autophagosomes and lysosomes. Autophagy is dependent on proteins such as the microtubule-associated protein light chain 3 (LC3) (Wilburn et al., 2021). SMW is associated with metabolic reprogramming, characterized by increased lactate production and decreased ATP production (Constantinou et al., 2011; Masi and Patel, 2021; Tzika et al., 2013). In addition, increased apoptosis in skeletal muscle is associated with SMW (Baltgalvis et al., 2010; de Castro et al., 2019; Miao et al., 2021). Despite the well-documented roles of tumor necrosis factors, transforming growth factors and interleukins in promoting cancer-associated SMW, clinical trials targeting these molecules have been unsuccessful in treating SMW (Biswas and Acharyya, 2020), indicating that other factors may compensate for their loss.

Chemokines are small soluble factors (8 kda) that form molecular gradients to regulate homing of immune cells to tissues during inflammation. They are subdivided into C-C, CXC and CX3C classes depending on the composition of a conserved cysteine motif. Chemokines bind to seven-transmembrane-spanning receptors, signaling through G protein-dependent and -independent mechanisms to enhance cell migration (Kohli et al., 2022). The chemokine CCL2 is a member of the C-C class of chemokines and is an important regulator of myeloid cells and T cells. Although CCL2 can bind multiple receptors, it binds with the highest affinity to CCR2 to modulate intracellular signaling and cell function (Fei et al., 2021). There is controversy regarding the role of CCL2–CCR2 signaling in skeletal muscle. CCL2-deficient mice display defects in muscle regeneration (Ferrara et al., 2023); yet, inhibition of CCR2 signaling aids muscle recovery from injury (Blanc et al., 2020). Increased CCL2 expression has been detected in serum and muscle tissues in patients with Duchenne's muscular dystrophy (Ogundele et al., 2021). In pancreatic, colon and breast cancers, CCL2 and CCR2 overexpression correlates with unfavorable prognosis. In patients with pancreatic cancer, elevated CCL2 levels in circulating blood were associated with cachexia (Talbert et al., 2018). Elevated levels of CCL2 in peripheral blood indicate possible systemic effects of this chemokine in patients with breast cancer (Fei et al., 2021; Hao et al., 2020). Currently, a role for CCL2–CCR2 signaling in SMW associated with cancer remains poorly understood. Using *in vitro* and *in vivo* models, this study identifies an important role for CCL2–CCR2 signaling in SMW associated with breast cancer, with important implications for therapeutic targeting.

## RESULTS

### CCL2 inhibits myoblast and myotube function in a concentration-dependent manner

**Fig. 1.**
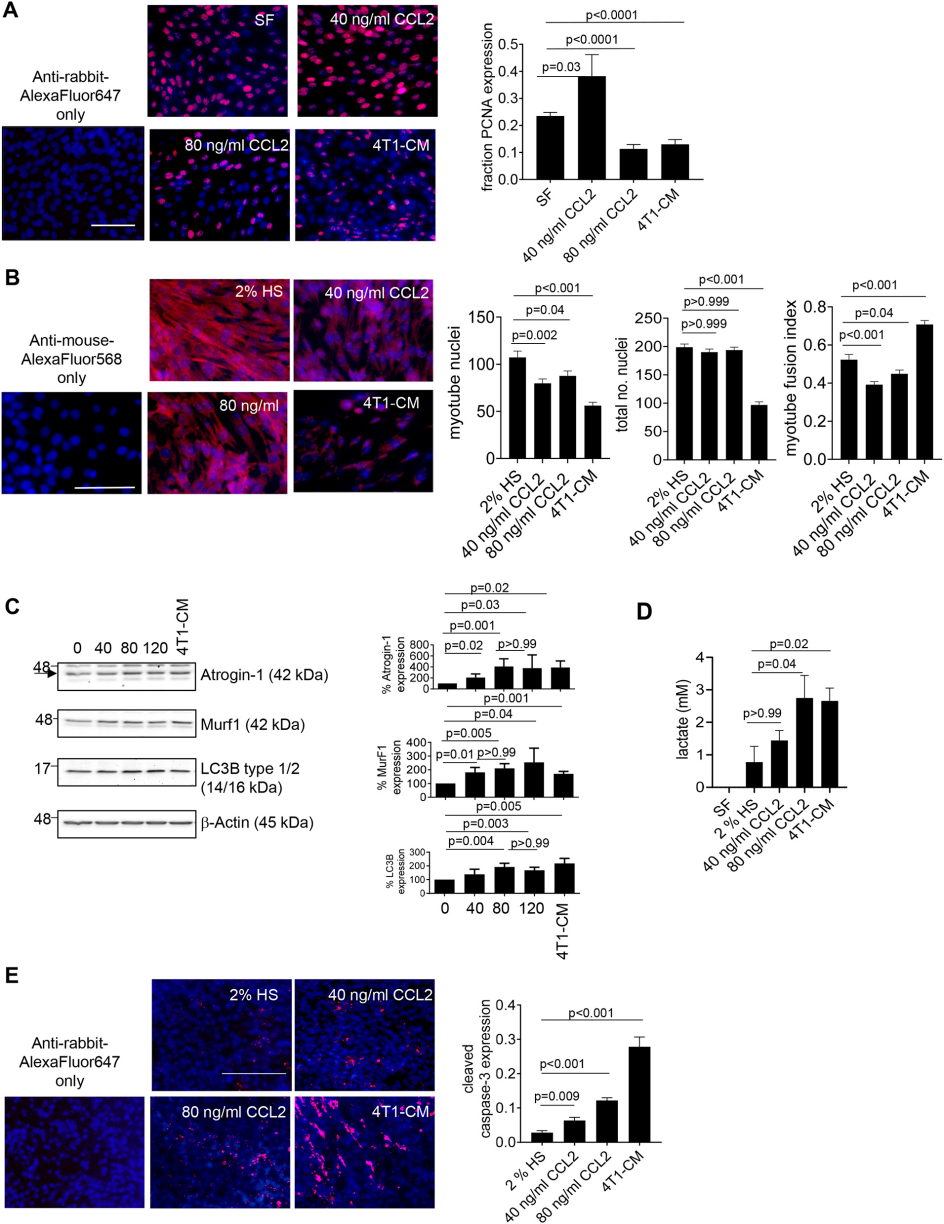
**CCL2 inhibits activity in myoblasts and myotubes at high concentrations.** (A,B) C2C12 myoblasts were treated with serum-free (SF) DMEM or DMEM containing 2% horse serum (HS), 40 or 80 ng/ml CCL2, or 4T1 tumor conditioned medium (4T1-CM) for 24 h and analyzed for proliferation by PCNA immunostaining (red) (A), or analyzed for myotube formation through the assessment of myotube fusion index (using an antibody against myosin heavy chain or MYH, red) for up to 96 h (B). (C-E) C2C12 myotubes were treated with SF medium, DMEM containing 2% HS, 40 or 80 ng/ml CCL2, or 4T1-CM. (C) Samples were analyzed for expression of the indicated proteins by immunoblotting. Densitometry was performed on immunoblots. Band intensities of samples were normalized to those of actin and expressed as a percentage relative to those of untreated samples. The arrow indicates the bands analyzed. (D) Intracellular lactate production was measured by biochemical assay. Lactate levels in SF samples were below the limit of detection. (E) The degree of apoptosis was analyzed by cleaved caspase-3 immunostaining. For A,B,E, biomarker expression was quantified by ImageJ (arbitrary units); samples were plated in triplicate and experiments were repeated three times (*n*=9/group). For C, experiments were repeated five times (*n*=5/group). For D, samples were plated in triplicate and performed four times (*n*=12/group). Statistical analysis was performed using one-way ANOVA with Bonferroni’s post hoc comparison (A,B,D,E) or Kruskal–Wallis test with Wilcoxon rank sum post hoc analysis (C). Relevant post hoc comparisons are indicated by lines. Significance was determined by *P*<05. Means±s.e.m. are shown. Scale bars: 100 μm.

As breast tumor tissues and cells chronically overexpress CCL2 (Fang et al., 2012, 2021), we hypothesized that increased CCL2 levels would adversely affect muscle cells. To determine this, we examined the effects of increasing concentrations of CCL2 on C2C12 murine myoblasts and myotube cultures. Myoblast proliferation was enhanced by a low CCL2 concentration (40 ng/ml) and inhibited with a higher concentration (80 ng/ml) (Fig. 1A). To examine myotube formation, we measured the myotube fusion index. Compared to that in untreated cells, low and high concentrations of CCL2 reduced the myotube fusion index, associated with a reduced number of myotube nuclei (Fig. 1B). In established myotube cultures, treatment with increasing CCL2 concentrations enhanced the expression of atrogin-1, MuRF1 and LC3B (encoded by *Map1lc3b*). There was no notable difference in protein expression with 80 or 120 ng/ml CCL2, suggesting a possible saturation of CCR2 binding with CCL2 at 80 ng/ml (Fig. 1C). High CCL2 increased lactate production in myotube cultures, but not ATP production (Fig. 1D; Fig. S1). These phenotypes were associated with increased cellular apoptosis but not necrosis as indicated by increased cleaved caspase-3 expression and annexin-V/propidium iodide staining. In contrast, low CCL2 did not significantly affect apoptosis or necrosis (Fig. 1E; Fig. S2). Overall, high CCL2 concentrations inhibit myoblast proliferation and myotube formation and, in myotubes, increase expression of SMW biomarkers, enhance lactate production and increase apoptosis.

To examine the relevance of CCL2 in breast cancer, we examined the effects of conditioned medium from metastatic 4T1 mammary carcinoma cells. Treatment with 4T1 tumor conditioned medium (4T1-CM) inhibited myoblast proliferation (Fig. 1A). 4T1-CM treatment resulted in a higher myotube fusion index compared to that in untreated cells (Fig. 1B). However, this was associated with a significant reduction in the number of total nuclei, likely from reduced proliferation and/or increased cell death during the differentiation process. Furthermore, we observed a reduction in the number of myotube nuclei, indicating decreased myotube formation with 4T1-CM treatment. In myotube cultures, 4T1-CM treatment increased the expression of muscle-wasting markers, increased lactate production to similar levels as 80 ng/ml CCL2 treatment, and increased apoptosis (Fig. 1A-E). Similarly, treatment with conditioned medium from MDA-MB-231 metastatic breast cancer cells (MDA-MB-231-CM) reduced myoblast proliferation, increased lactate production and increased apoptosis in myotube cultures. Treatment with MDA-MB-231-CM resulted in a higher myotube fusion index, which was associated with a significantly reduced number of total nuclei and a moderate increase in the number of myotube nuclei (Fig. S3A-D). Treatment with conditioned media from both cell lines inhibited ATP production in myotube cultures (Fig. S1). Overall, breast tumor conditioned media inhibit myoblasts and myotubes, with some similar effects to those of high CCL2 treatment.

**Fig. 2.**
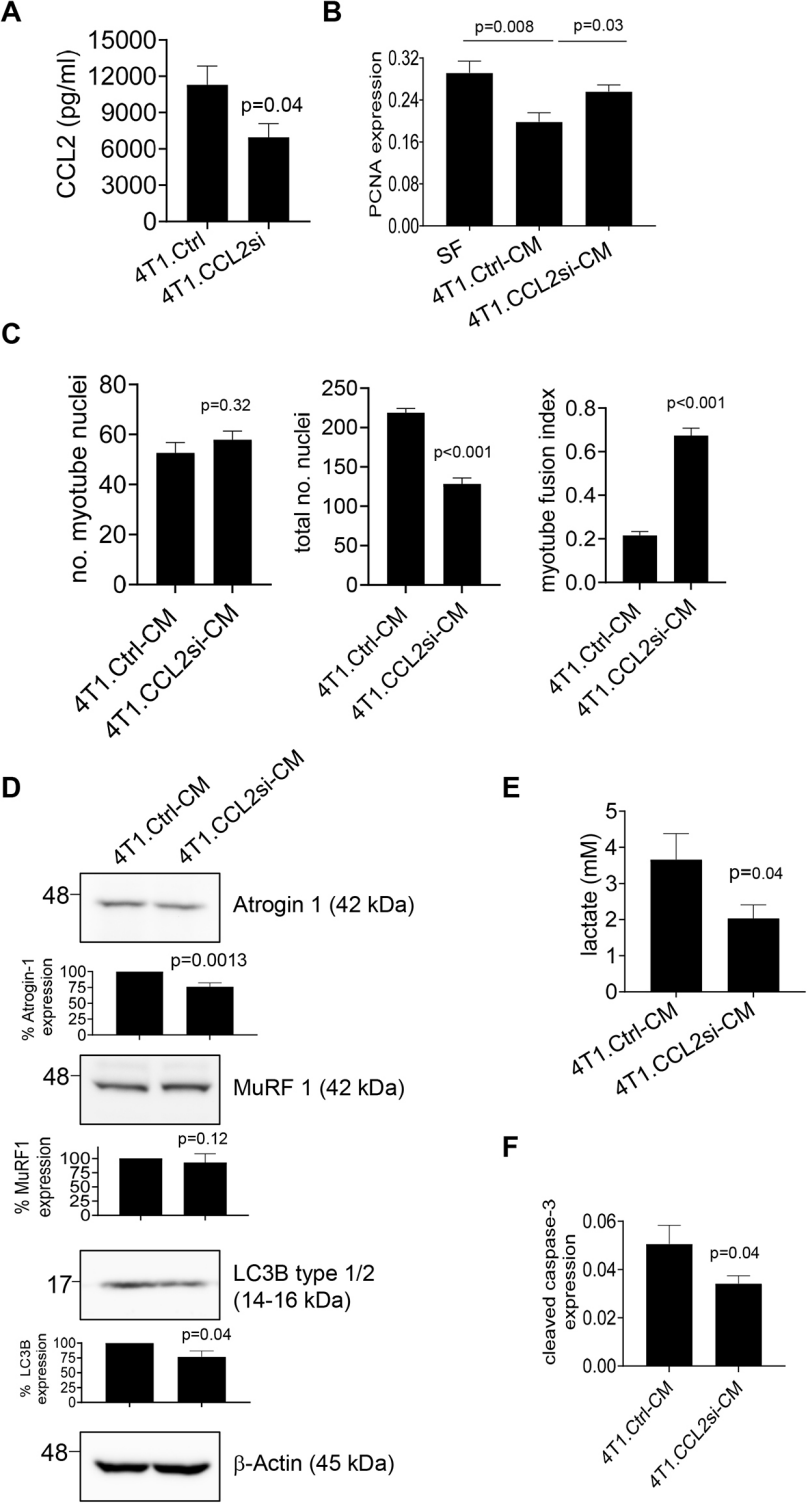
**CCL2 knockdown prevents mammary carcinoma cells from inhibiting myoblast and myotube activity.** (A) 4T1 cells were transfected with control siRNA (4T1.Ctrl) or CCL2 siRNA (4T1.CCL2si) and analyzed for CCL2 levels by ELISA. (B,C) C2C12 myoblasts were treated with serum-free (SF) medium or with conditioned medium from 4T1 cells transfected with control siRNA (4T1.Ctrl-CM) or CCL2 siRNA (4T1.CCL2si-CM) for 24 h and analyzed for cell proliferation by PCNA immunostaining (B) or for differentiation into myotubes through assessment of myotube fusion index (C). (D) C2C12 myotubes were treated with tumor conditioned medium and analyzed for changes in the expression of atrogin-1, MuRF1 and LC3B by immunoblotting. Densitometry was performed on immunoblots. Band intensities of samples were normalized to those of actin and expressed as a percentage relative to those of 4T1.Ctrl-CM samples (D). (E) Intracellular lactate production was measured by biochemical assay. (F) The degree of apoptosis was determined by cleaved caspase-3 immunostaining. For A-C,E,F, biomarker expression was quantified by ImageJ (arbitrary units); samples were plated in triplicate and experiments were performed three times (*n*=9/group). For D, experiments were repeated five times (*n*=5/group). Statistical analysis was performed using two-tailed unpaired *t*-test (A,E,F), one-way ANOVA with Bonferroni's post hoc comparison (B,C) or Wilcoxon rank sum test (D). For *P*<0.05 determined by ANOVA, relevant post hoc comparisons are indicated by lines. Statistical significance was defined by *P*<0.05. Means±s.e.m. are shown.

Previous studies have indicated that 4T1 cells express CCL2 (Fang et al., 2012). In this study, CCL2 levels were significantly lower in C2C12 cells compared to in 4T1 cells (Fig. S4A). To determine the contribution of tumor cell-derived CCL2 to myoblast and myotube activity, CCL2 was knocked down by siRNA or shRNA in 4T1 cells, resulting in a respective 47% and 70% reduction (Fig. 2A; Fig. S4A). C2C12 myoblasts were then treated with conditioned medium from 4T1 cells transfected with Ctrl siRNA (4T1.Ctrl-CM) or CCL2 siRNA (4T1.CCL2si-CM), or with 4T1 cells stably expressing control EGFP shRNA (4T1.EGFPsh-CM) or CCL2 shRNA (4T1.CCL2sh-CM). 4T1.CCL2si-CM or 4T1.CCL2sh-CM treatment increased myoblast proliferation and, in myotubes, decreased atrogin-1 and LC3B expression, lactate production and cellular apoptosis. Differing effects were observed for myotube formation. The myotube fusion index was reduced following treatment with control medium (4T1.Ctrl-CM) and increased following treatment with 4T1.CCL2si-CM. In contrast, conditioned medium from EGFP shRNA- and CCL2 shRNA-expressing 4T1 cells resulted in a higher myotube fusion index, associated with a decreased total number of nuclei from control and CCL2-deficient groups (Fig. 2B-F; Fig. S4B-F). Overall, CCL2 siRNA knockdown enhanced myoblast proliferation and myotube formation and, in myotubes, reduced the expression of SMW biomarkers and decreased lactate production and apoptosis. Except for myotube formation, stable CCL2 shRNA knockdown resulted in similar effects to those for siRNA knockdown on myoblast and myotube cultures.

### CCL2–CCR2 signaling inhibits myoblast and myotube function through SMAD3-, JNK- and AMPK-dependent mechanisms

**Fig. 3.**
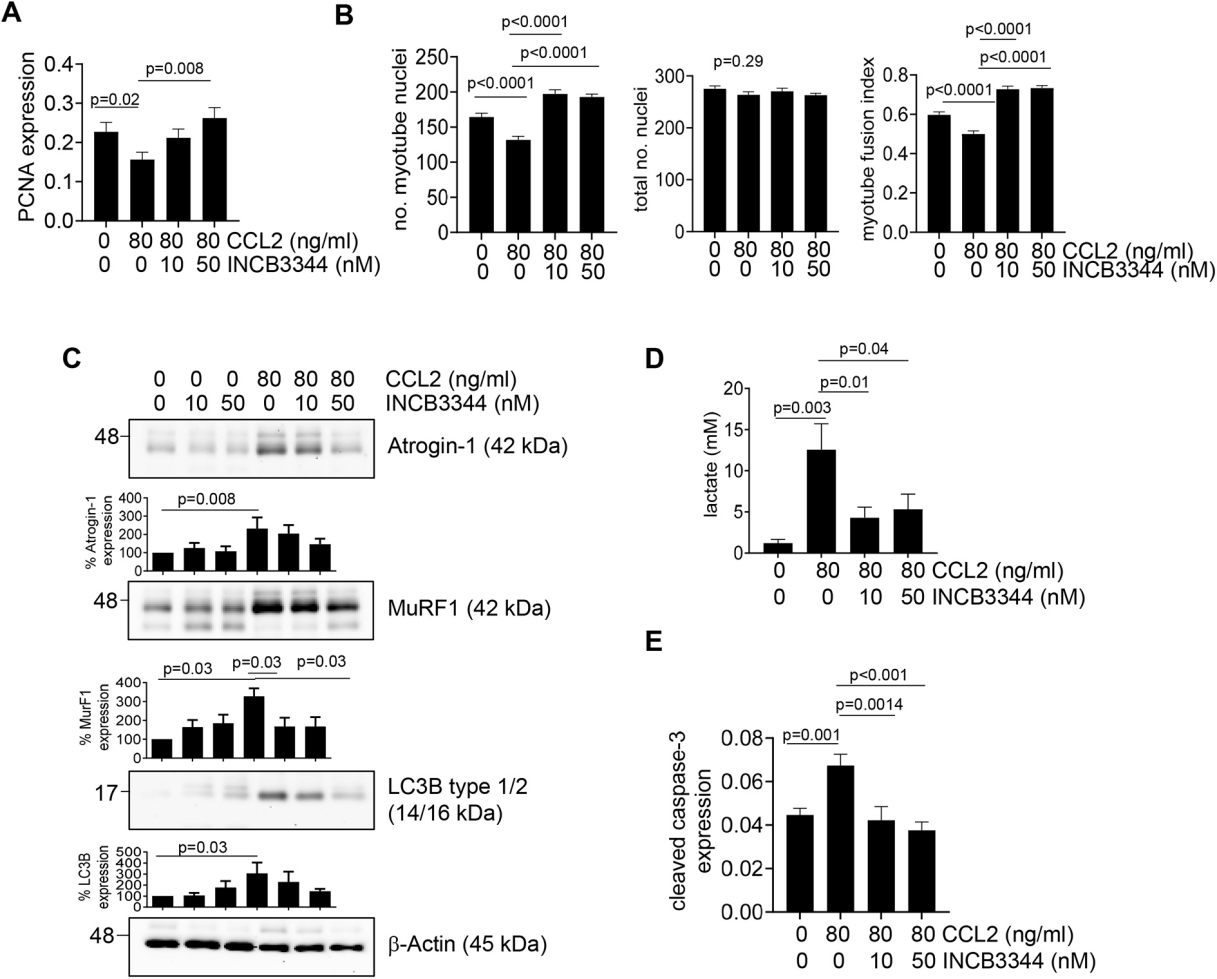
**CCR2 inhibition by INCB3344 treatment rescues myoblast proliferation, myotube formation and myotube function.** (A,B) C2C12 myoblasts were treated with 80 ng/ml CCL2 with increasing concentrations of INCB3344 for 24 h and analyzed for cell proliferation by PCNA immunostaining (A) or for myotube formation through the assessment of myotube fusion index (B). (C) C2C12 myotube cultures were treated with CCL2 with or without INC3344 and analyzed for the expression of atrogin-1, MuRF1 or LC3B by immunoblotting. Densitometry was performed on immunoblots. Band intensities of samples were normalized to those of actin and expressed as a percentage relative to those of untreated cells. (D) Intracellular lactate production was measured by biochemical assay. (E) The degree of apoptosis was determined by cleaved caspase-3 immunostaining. Biomarker expression was quantified by ImageJ (arbitrary units). For A,B,D,E, samples were plated in triplicate and experiments were performed three times (*n*=9/group). For C, experiments were repeated five times (*n*=5/group). Statistical analysis was measured using one-way ANOVA with Bonferroni’s post hoc comparison (A,B,D,E) or Kruskal–Wallis test with Wilcoxon rank sum post hoc analysis (C). Statistical significance was defined as *P*<0.05. For *P*>0.05 determined by ANOVA, the *P*-value is shown in the top left corner of the graph. For *P*<0.05 determined by ANOVA, relevant post hoc comparisons are indicated by lines. Means±s.e.m. are shown.

We determined the effects of treatment with CCL2 or 4T1-CM on CCR2 expression, the primary receptor for CCL2, in skeletal muscle cells. Reduced CCR2 expression in muscle is associated with decreased adverse effects from tumor growth in mice (Esmailiyan et al., 2023). By immunofluorescence and flow cytometry, treatment with 80 ng/ml CCL2 or 4T1-CM enhanced CCR2 expression in C2C12 myoblasts but not in myotubes (Fig. S5A-C). We examined the functional significance of elevated CCR2 expression by blocking its activity in C2C12 cells with the receptor antagonist INCB3344 (Xue et al., 2010). In myoblasts treated with high levels of CCL2, INCB3344 treatment rescued cell proliferation and myotube formation (Fig. 3A,B). In myotube cultures, INCB3344 treatment significantly reduced CCL2-induced MuRF1 expression, lactate production and cellular apoptosis (Fig. 3C-E). Overall, CCR2 is important for CCL2-mediated inhibition of myoblast proliferation and differentiation, and enhancement of myotube expression of MuRF1, lactate production and apoptosis.

To understand how intracellular signaling facilitated the CCL2-mediated inhibition of myoblast and myotube activities, we performed a candidate screening of known CCL2–CCR2 signaling targets, including SMAD3, p42/p44 MAPKs (encoded by *Mapk1* and *Mapk3*, respectively) and SRC (Fang et al., 2012; Yao et al., 2019). We examined the changes in the levels of phosphorylated JNK proteins (encoded by *Mapk8*, *Mapk9* and *Mapk10*) and AMPK-α (encoded by *Prkaa1*), pathways associated with SMW (Mulder et al., 2020; White et al., 2013). By immunoblotting, high CCL2 treatment of myoblasts decreased phosphorylation of SRC and p42/p44 MAPKs, consistent with decreased mitogenic activity. High CCL2 treatment also increased phosphorylation of JNK, SMAD3 and AMPK-α in myoblasts (Fig. S6). In myotube cultures, high CCL2 treatment decreased phosphorylation of SRC, increased phosphorylation of JNK, AMPK-α and SMAD3, and did not affect p42/p44 MAPK phosphorylation (Fig. S6). SMAD3 associates with FOXO transcription factors and negatively regulates peroxisome proliferator-activated receptors (PPARs) to modulate gene expression and muscle activity (Bollinger et al., 2014; Goodman et al., 2013; Tiano et al., 2015). Therefore, we determined whether CCL2 also affected expression of FOXO proteins, PPARγ (encoded by *Pparg*) and the associated co-activator PGC1α (encoded by *Ppargc1a*) in myoblast and myotube cultures. By immunoblotting, CCL2 (80 ng/ml) treatment significantly increased phosphorylation of FOXO3 in myoblasts but not in myotube cultures, and decreased expression of PPARγ and PGC1α over time (Fig. S7A). By co-immunofluorescence staining of myoblasts, CCL2 treatment increased nuclear colocalization of SMAD3 and FOXO3 over time (Fig. S7B). Thus, CCL2 enhances multiple pathways in myoblasts and myotubes including JNK, AMPK and SMAD3–FOXO3 activity, and downregulates PPARγ.

**Fig. 4.**
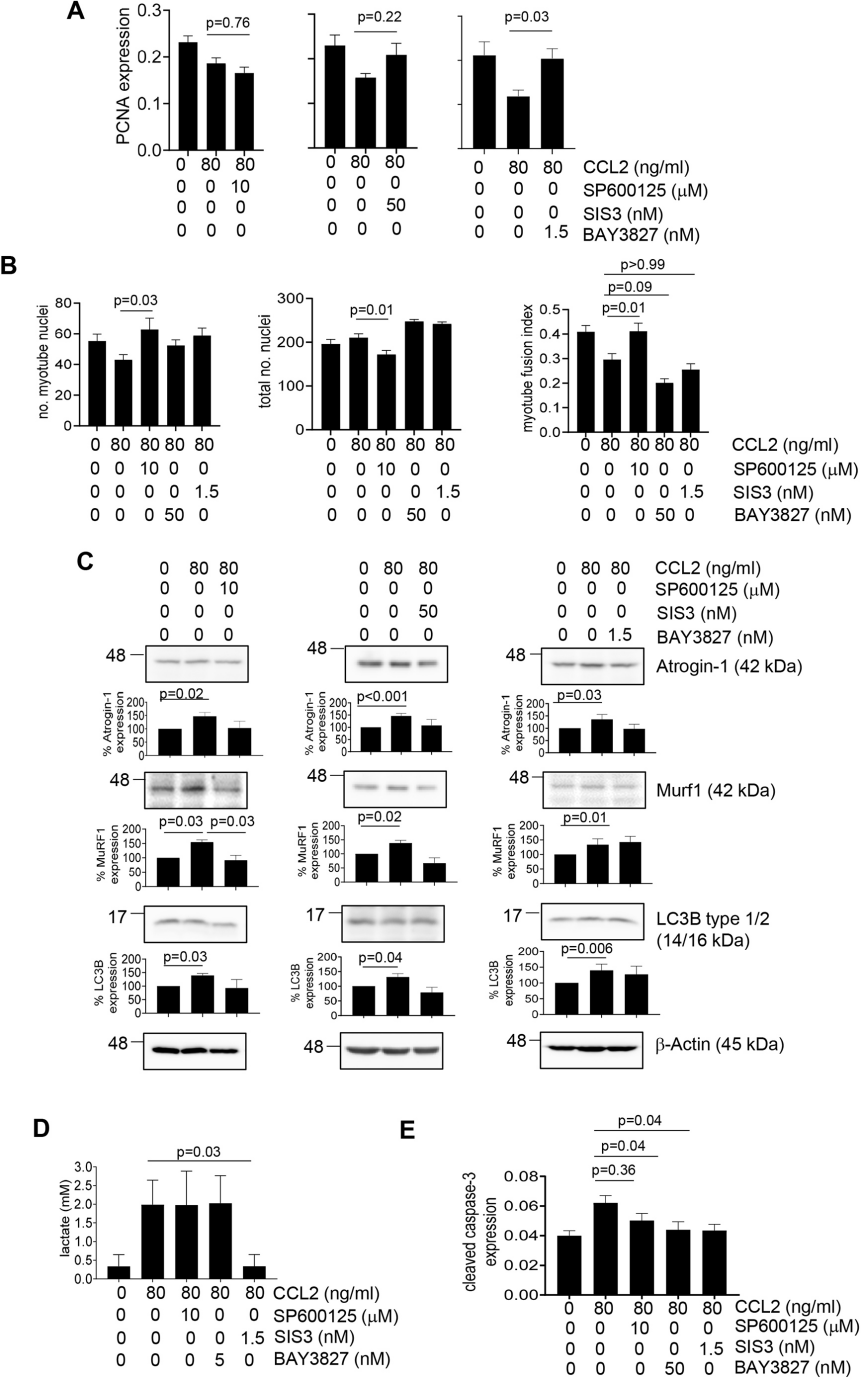
**CCL2 negatively regulates myoblast proliferation, myotube formation and myotube activity through JNK-, SMAD3- and AMPK-dependent mechanisms.** (A,B) C2C12 myoblasts were treated with 80 ng/ml CCL2 in the presence or absence of 10 mM SP600125 (JNK inhibitor), 50 nM SIS3 (SMAD3 inhibitor) or 1.5 mM BAY3827 (AMPK inhibitor) and analyzed for changes in proliferation by PCNA immunostaining (A) or for myotube formation through the assessment of myotube fusion index (B). (C) C2C12 myotubes were treated with 80 ng/ml CCL2 in the presence or absence of SP600126, SIS3 or BAY3827 and analyzed for expression of the indicated proteins by immunoblotting. Densitometry was performed on immunoblots. Band intensities of samples were normalized to those of actin and expressed as a percentage relative to those of untreated cells. (D) Intracellular lactate production was measured by biochemical assay. (E) The degree of apoptosis was determined by cleaved caspase-3 immunostaining. Biomarker expression was quantified by ImageJ (arbitrary units). For A,B,D,E, samples were plated in triplicate and experiments were performed three times (*n*=9/group). For C, experiments were repeated five times (*n*=5/group). Statistical analysis was performed using one-way ANOVA with Bonferroni's post hoc comparison (A,B,D,E) or Kruskal–Wallis test with Wilcoxon rank sum post hoc analysis (C). Relevant post hoc comparisons are indicated by lines. Statistical significance was determined by *P*<0.05. Means±s.e.m. are shown.

Using pharmacological inhibitors, we determined the significance of these pathways to CCL2-mediated inhibition of myoblasts and myotubes. Inhibitory concentrations of the JNK inhibitor SP600125, the SMAD3 inhibitor SIS3 and the AMPK inhibitor BAY3827 were determined by immunoblotting and cell viability assays (Fig. S8A-F). In myoblasts, CCL2 treatment with BAY3827 but not SIS3 or SP600125 enhanced cell proliferation over treatment with CCL2 alone (Fig. 4A). CCL2 treatment with SP600125 but not SIS3 or BAY3827 enhanced the myotube fusion index associated with increased numbers of myotube nuclei and reduced the total number of nuclei (Fig. 4B). In myotube cultures, only SP600125 treatment significantly inhibited CCL2-induced expression of MuRF1. Only BAY3827 treatment inhibited CCL2-induced lactate production (Fig. 4C,D). CCL2-induced apoptosis was inhibited by treatment with SIS3 and BAY3827 but not with SP600125 (Fig. 4E). Overall, these data indicate that, in myoblasts, AMPK is important in CCL2-mediated inhibition of cell proliferation, whereas JNK is important in CCL2-mediated inhibition of myotube formation. In myotubes, JNK mediates CCL2 inhibition of MuRF1, AMPK regulates CCL2-induced lactate production, and SMAD3 and AMPK modulate CCL2-induced apoptosis.

### CCL2 delivery *in vivo* affects lean mass and muscle strength in a dose-dependent manner

**Fig. 5.**
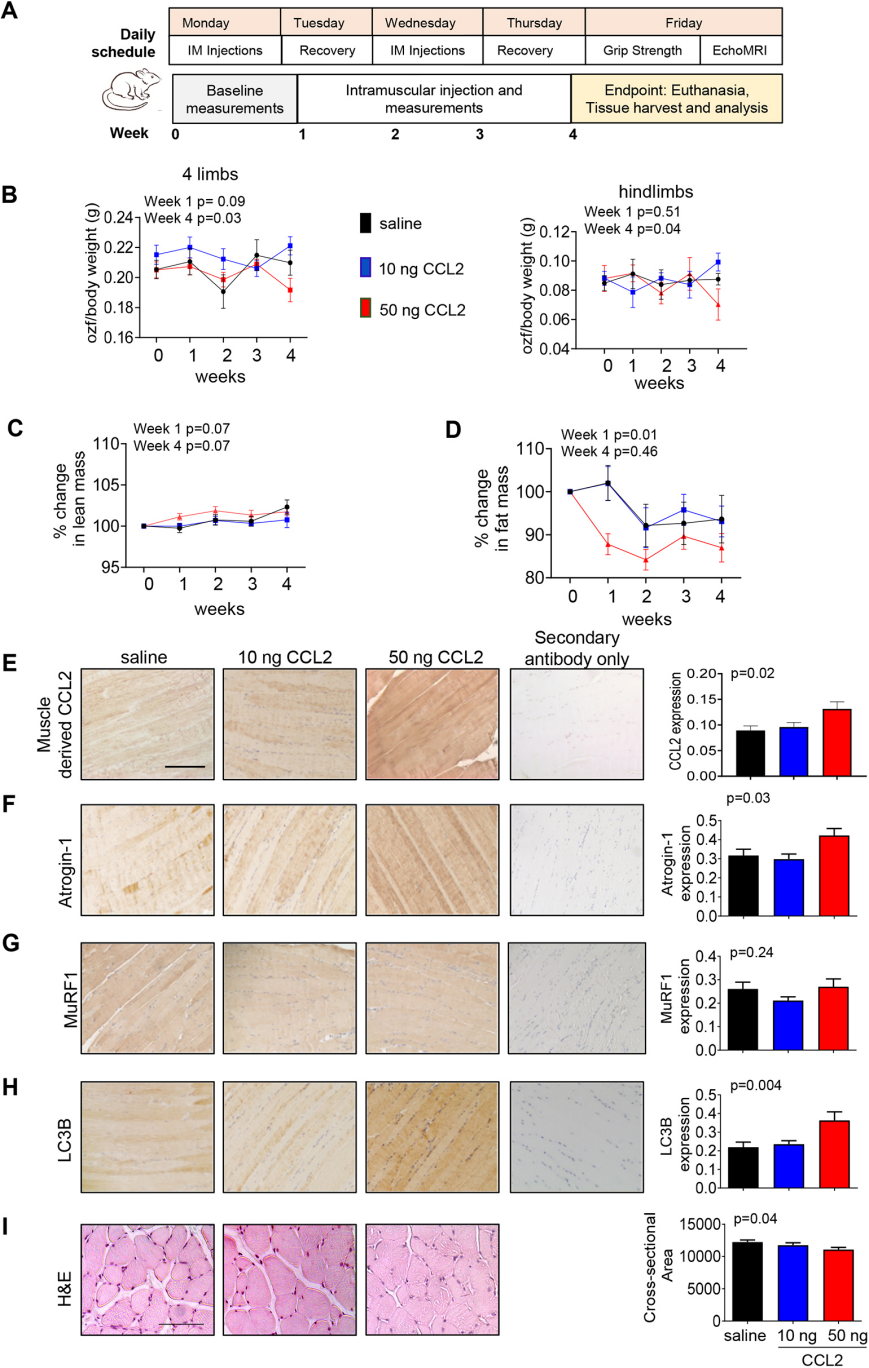
***In vivo* delivery of high-dose CCL2 reduces grip strength and fat mass.** (A) Daily schedule of CCL2 delivery and assessment of muscle strength and body mass composition. Healthy, tumor-free, female BALB/c mice received intramuscular (IM) injections of recombinant murine CCL2 (10 or 50 ng) or saline vehicle control twice a week (*n*=9/group). Mice were then subjected to grip strength tests and EchoMRI once a week, the day after the second intramuscular injection. Baseline measurements were taken at week 0. Mice were assessed during weeks 1-4 and then euthanized for tissue harvest. (B) Grip strength normalized to body mass is shown for all four limbs (left graph) and hindlimbs (right graph). Grip strength units are defined by ounce-force (ozf). (C,D) Lean mass (C) and fat mass (D) were assessed by EchoMRI. Lean and fat masses relative to body weight were normalized to baseline measurements (week 0). (E-H) Quadricep muscle tissues were immunostained for CCL2 (E), atrogin-1 (F), MuRF1 (G) or LC3B (H). Longitudinal sections are shown. (I) Analysis of cross-sectional area of quadricep muscle tissues stained with Hematoxylin and Eosin (H&E). Biomarker expression or cross-sectional area was quantified using ImageJ (arbitrary units). Statistical analysis was performed using one-way ANOVA with Bonferroni's post hoc comparison. Statistical significance was defined by *P*<0.05. For B-D, the *P*-value determined by ANOVA for weeks 1 and 4 is shown on the top left corner of each line graph. For E-I, the *P*-value determined by ANOVA is shown on the top left corner of each bar graph. Means±s.e.m. are shown. Scale bars: 100 μm.

Given the adverse effects of high CCL2 treatment on myoblasts and myotubes *in vitro*, we determined whether the delivery of recombinant CCL2 protein adversely affected skeletal muscle tissues *in vivo*. Based on studies involving *in vivo* delivery of recombinant CCL2 (Fang et al., 2021), we delivered CCL2 at a low dosage (10 ng) or high dosage (50 ng) to female BALB/c mice via intramuscular injection. After twice-weekly injections, mice were tested for grip strength and measurements of body mass composition using the EchoMRI system for up to 4 weeks (Fig. 5A). At the endpoint, 50 ng CCL2 treatment was associated with reduced grip strength in all four limbs and in hindlimbs, whereas 10 ng CCL2 treatment was associated with higher grip strength (Fig. 5B; unnormalized data shown in Fig. S9A). 50 ng CCL2 treatment was also associated with a transient decrease in fat mass after 1 week, but not at endpoint. Lean mass did not change among groups (Fig. 5C,D; unnormalized data shown in Fig. S9B). Statistical comparisons for grip strength and body mass are summarized in Table S1. Overall, 50 ng CCL2 treatment decreases muscle strength and transiently reduces fat mass.

We immunostained skeletal muscle tissues for CCL2, atrogin-1, MuRF1 and LC3B expression. Biomarker expression was quantified by ImageJ, a commonly used approach to quantify biomarker expression in various tissue types including tumor and muscle (Brummer et al., 2020; Fang et al., 2021, 2016; Guirado et al., 2018; Minagawa et al., 2023; Yao et al., 2016). ImageJ quantification of biomarker expression correlates with the clinical method of manual scoring and provides continuous values to facilitate a more thorough statistical analysis of data (Fang et al., 2021). Overall, we detected a significant increase in CCL2 expression in muscle tissue with 50 ng CCL2 treatment, associated with elevated atrogin-1 and LC3B but not MuRF1 expression compared to their expression in muscle tissue with vehicle control or 10 ng CCL2 treatment (Fig. 5E-H). Statistical comparisons for biomarker expression are summarized in Table S2.

We then analyzed skeletal muscle size and composition. 50 ng CCL2 treatment was associated with reduced cross-sectional area and Feret diameter of muscle fibers in mice compared to those in control mice (Fig. 5I; Fig. S10A). The composition of muscle fibers was assessed through immunohistochemical analysis of myosin heavy chain (MYH) and myosin light chain (MYL). Compared to saline control treatment, 50 ng CCL2 treatment did not significantly affect the expression of total MYH or MYL (Fig. S10B,C). Fast twitch biomarker expression, notably that of MYH4 (de Wilde et al., 2010; Dos Santos et al., 2022), was examined in quadricep muscle. Compared to saline control, 50 ng CCL2 treatment significantly reduced the expression of MYH4 in muscle tissues. 10 ng CCL2 treatment significantly increased MYL expression and but did not affect MYH4 expression over their expression levels with saline control treatment (Fig. S10D,E). Statistical comparisons for myofiber size and biomarker expression are summarized in Tables S2 and S3. Overall, 50 ng CCL2 delivery reduces the expression of MYH proteins.

### CCL2 knockdown in mammary tumor-bearing mice alters body mass composition and reduces expression of muscle-wasting biomarkers

Given the inhibitory effects of tumor-derived CCL2 on myoblasts and myotubes *in vitro*, we determined how targeting of CCL2 expression in breast tumors would affect SMW *in vivo* by knocking down CCL2 through the delivery of TAT cell-penetrating peptides with calcium cross-links to siRNAs (Ca-TAT/siRNAs). These peptide–siRNA complexes were shown to effectively penetrate mammary tumor tissues and induce gene knockdown in mice (Brummer et al., 2020; Fang et al., 2016). Ca-TAT/siRNA complexes were delivered via intra-tumoral injection, approximately 7 days after orthotopic transplantation of 4T1 cells.

**Fig. 6.**
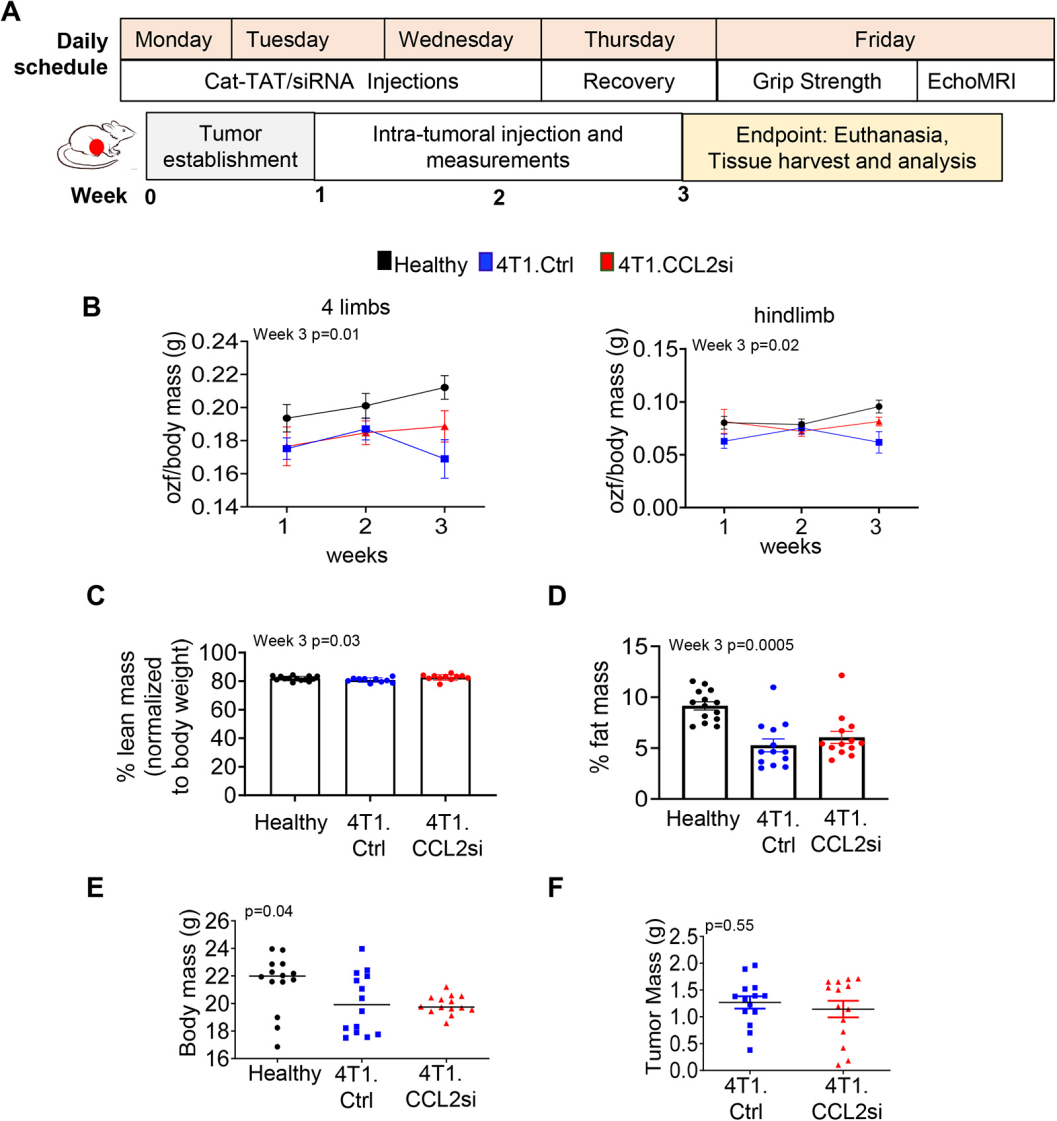
**Tumoral CCL2 siRNA knockdown rescues lean mass but not grip strength.** (A) Daily schedule of Ca-TAT/siRNA delivery and assessment of muscle strength and body mass composition. BALB/c female mice bearing 4T1 mammary carcinomas received intra-tumoral injections of 10 µg Ca-TAT peptides complexed to control (Ctrl, *n*=13) or CCL2 (CCL2si, *n*=13) siRNAs, once a day for 3 days. Mice were subject to grip strength tests and EchoMRI one day after the last injection of Ca-TAT/siRNA complexes. Tumors were established at week 0. Intra-tumoral injections were performed during weeks 1 to 3, and then mice were euthanized for tissue harvest. Healthy normal BALB/c mice were used as a baseline control (*n*=14). (B) Grip strength normalized to body mass is shown for all four limbs (left) and hindlimbs (right). Grip strength units are defined by ounce-force (ozf). (C,D) Lean mass (C) and fat mass (D) were assessed by EchoMRI. The percentages of lean mass and fat mass were calculated by dividing the lean or fat mass by total body mass and multiplying by 100. (E,F) Total body mass (E) or tumor mass (F) at endpoint is shown for the indicated groups. Healthy, untreated mice were used as a baseline control (*n*=13). Statistical analysis was performed using one-way ANOVA with Bonferroni's post hoc analysis (B-E) or two-tailed unpaired *t*-test (F). For B-F, the *P*-value determined by ANOVA is shown on the top left corner of each graph. Statistical significance was defined by *P*<0.05. Means±s.e.m. are shown.

Tumor-bearing mice were treated three times a week with Ca-TAT/siRNA complexes, followed by measurements of grip strength and body mass composition (Fig. 6A). Healthy, non-tumor-bearing mice were used as a baseline control. Mice were assessed for 3 weeks, when control tumors reached 1.5 cm in diameter. Compared to normal healthy mice, 4T1 mammary tumor-bearing mice receiving control siRNAs (Ctrl) showed significantly reduced grip strength and fat mass, but not lean mass (Fig. 6B-D; unnormalized data shown in Figs S6B and S7C,D). Compared to mammary tumor-bearing mice receiving Ca-TAT/control siRNA (4T1.Ctrl), Ca-TAT/CCL2 siRNA (4T1.CCL2si) treatment enhanced lean mass and did not affect grip strength, fat mass or overall body mass (Fig. 6B-E; unnormalized data shown in Fig. S11A-C). CCL2 siRNA treatment did not significantly affect tumor mass (Fig. 6F), indicating that the effects of tumoral CCL2 knockdown on lean mass were independent of tumor growth. Statistical comparisons for grip strength and body mass are summarized in Table S4.

**Fig. 7.**
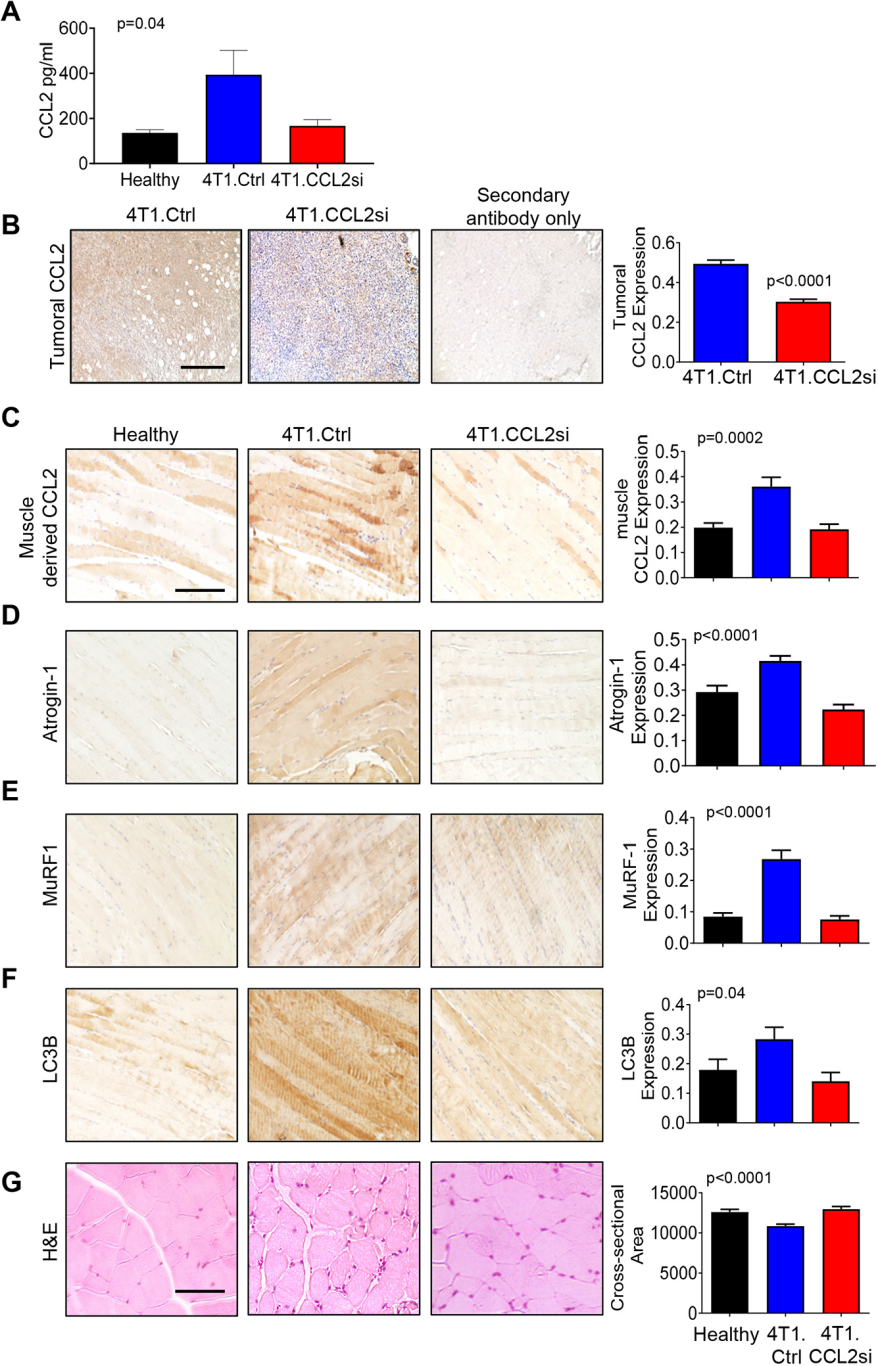
**Intra-tumoral knockdown of CCL2 reduces expression of biomarkers associated with skeletal muscle atrophy.** (A) Blood serum levels of CCL2 from mice at endpoint were measured by ELISA. (B) Immunostaining was performed for CCL2 in mammary epithelial tissues. (C-F) Immunostaining was performed for CCL2 (C), atrogin-1 (D), MuRF-1 (E) or LC3B (F) in quadricep muscle tissues. Longitudinal sections are shown. (G) The cross-sectional area was analyzed for quadricep muscle stained with H&E. Representative images from eight mice per group are shown. Biomarker expression or cross-sectional area was quantified using ImageJ (arbitrary units). Statistical analysis was measured using two-tailed unpaired *t*-test (A) or one-way ANOVA with Bonferroni's post hoc comparison (B-G). Statistical significance was defined *P*<0.05. For A,C-G, the *P*-value determined by ANOVA is shown on the top left corner of each graph. Means±s.e.m. are shown. Scale bars: 100 μm.

We analyzed the effects of CCL2 siRNA treatment on CCL2 and SMW biomarker expression. By enzyme-linked immunosorbent assay (ELISA), 4T1 tumor-bearing mice showed elevated CCL2 serum levels compared to healthy mice (mean±s.e.m.=393±108.4 pg/ml) (Fig. 7A), within range of those in patients with breast cancer (mean range=192-398 pg/ml) (Dwyer et al., 2007; Lubowicka et al., 2018). By immunostaining, 4T1 tumor-bearing mice showed increased CCL2 expression in tumors, corresponding to CCL2 levels in skeletal muscle tissue (Fig. 7B,C). CCL2 siRNA treatment partially reduced CCL2 serum levels and significantly reduced the expression of CCL2 in 4T1 mammary tumors and skeletal muscle tissues. The decreased CCL2 expression corresponded to reduced atrogin-1 MuRF1 and LC3B expression (Fig. 7D-F). Statistical comparisons for SMW biomarker expression are reported in Table S5. The effects of tumoral knockdown of CCL2 treatment on skeletal muscle tissue were further examined in the MDA-MB-231 human metastatic breast cancer model. CCL2 siRNA-mediated knockdown in MDA-MB-231 breast xenografts has been shown to inhibit primary tumor growth and metastasis (Fang et al., 2016). CCL2 knockdown was associated with reduced expression of atrogin-1, MurF-1 and LC3B in skeletal muscle tissue (Fig. S12), similarly to the 4T1 model. Overall, tumoral knockdown of CCL2 elevates lean mass and decreases expression of SMW markers.

We analyzed myofiber size and the expression of MYH and MYL proteins. Compared to healthy mice, 4T1 tumor-bearing mice showed reduced myofiber size and reduced expression of overall MYH but did not show significant changes in MYL or MYH4 expression (Fig. 7G; Fig. S13A-E). CCL2 siRNA treatment of 4T1 tumor-bearing mice increased myofiber size as indicated by measurements of cross-sectional area and Feret diameter, increased MYL expression, and reduced expression of total MYH but not MYH4 (Fig. 7G; Fig. S13A-E). Statistical comparisons for myofiber size and biomarker expression are reported in Tables S5 and S6. Overall, 4T1 tumor-bearing mice showed reduced myofiber size and MYH expression. CCL2 siRNA treatment enhanced myofiber size and altered the expression of MYH and MYL proteins, suggesting that other MYH isoforms are regulated by CCL2.

### Tumoral CCL2 knockdown alters the immune microenvironment in skeletal muscle

**Fig. 8.**
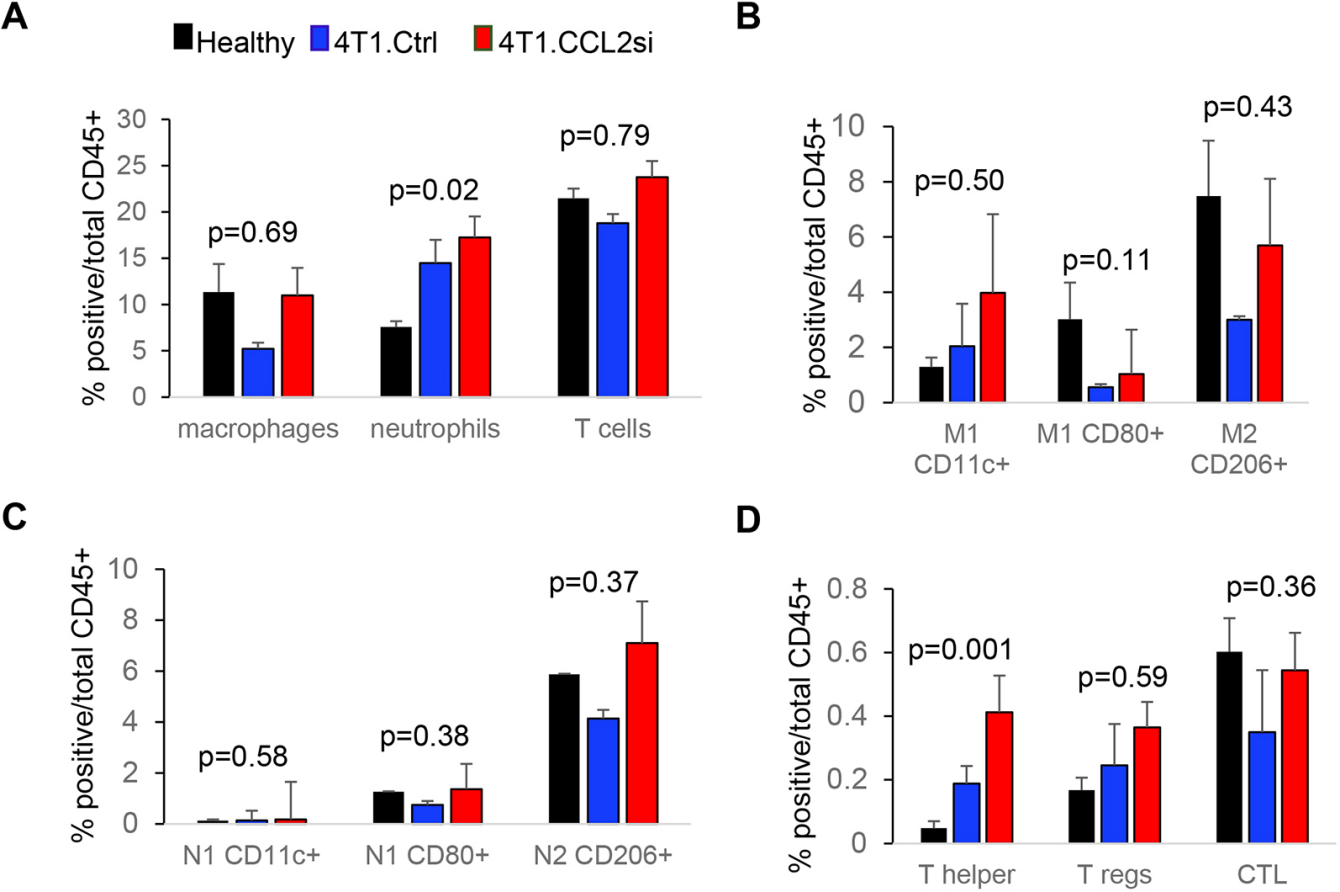
**Tumoral knockdown of CCL2 increases neutrophils and T helper cells in skeletal muscle tissues.** 4T1 tumor-bearing mice were treated with Ca-TAT peptides complexed to control (Ctrl) or CCL2 (CCL2si) siRNAs. At endpoint, gastrocnemius muscle tissues were harvested, digested into single-cell suspensions and analyzed by flow cytometry for the presence of (A) macrophages (F4/80^+^), neutrophils (F4/80^−^/Ly6G^+^) and T cells (CD3^+^); (B) macrophage subpopulations – M1 (F4/80^+^ CD11b^+^ Ly6C^+^ CD11c^+^ or F4/80^+^ CD11b^+^ Ly6C^+^ CD80^+^) or M2 (F4/80^+^ CD11b^+^ Ly6C^+^ CD206^+^); (C) neutrophil subpopulations – N1 (F4/80^−^ Ly6G^+^ CD11b^+^ CD11c^+^ or F4/80^−^ Ly6G^+^ CD11b^+^ CD80^+^) or N2 (F4/80^−^/Ly6G/CD11b/CD206); and (D) T cell subpopulations – T helper cells (CD3^+^ CD4^+^ CD25^−^), T regs (CD3^+^ CD4^+^ CD25^+^) or cytotoxic T lymphocytes (CTLs) (CD3^+^ CD8a^+^). *n*=8/group. Skeletal muscle tissues from healthy, tumor free mice were used as a baseline control. Statistical analysis was performed using one-way ANOVA with Bonferroni's post hoc comparison. Statistical significance was determined by *P*<0.05. For A-D, the *P*-value determined by ANOVA is shown for each immune group comparison. Means±s.e.m. are shown.

CCL2 is an important regulator of myeloid cell and T cell recruitment during inflammation and infection (Fei et al., 2021). Therefore, we determined whether tumoral knockdown of CCL2 might affect the immune microenvironment through flow cytometry analysis of skeletal muscle tissues. This approach allowed us to profile the expression of twelve immune biomarkers in tissues and obtain quantitative insight into myeloid and lymphoid immune subsets among healthy, 4T1.Ctrl- and 4T1.CCL2si-treated mice (Tables S7 and S8). We first measured the overall levels of macrophages, neutrophils and T cells in healthy untreated mice, Ctrl siRNA- and CCL2 siRNA-treated mice in the 4T1 model. There were no significant differences in the overall percentage of macrophages or T cells among groups. The percentage of neutrophils was significantly increased with CCL2 siRNA treatment compared to that in healthy untreated mice (Fig. 8A). Given the heterogeneity of myeloid cells and T cells, we analyzed for changes in immune subpopulations. For macrophages and neutrophils, we assayed for markers associated with classically activated cells (CD80^+^, Cd11c^+^) or wound healing (CD206^+^) (Holmberg-Thyden et al., 2021). For T cells, skeletal muscle tissues were analyzed for biomarkers associated with T helper cells (CD4^+^ CD25^−^), regulatory T cells (T regs) (CD4^+^ CD25^+^), cytotoxic T cells (CD8a^+^) (Holmberg-Thyden et al., 2021). There were no significant changes in macrophage or neutrophil subpopulations among groups (Fig. 8B,C). However, with CCL2 siRNA treatment, we observed a significant increase in CD4^+^ T cells compared to those in untreated mice. There were no changes in other T cell populations (Fig. 8D). Overall, tumoral knockdown of CCL2 increases the levels of neutrophils and CD4^+^ T cells in skeletal muscle tissues.

## DISCUSSION

Altogether, we present a model for how CCL2 functions during SMW. Breast tumoral secretion of CCL2 enhances its bioavailability in skeletal muscle tissues, increasing CCL2–CCR2 signaling and inhibiting myoblast and myotube activity through JNK-, AMPK- and SMAD3-dependent mechanisms. In differentiated cells, increased proteasomal degradation and autophagy and reduced survival lead to decreased muscle mass and diminished overall function. Decreased myoblast proliferation and differentiation prevent the formation of new skeletal muscle tissue and further impair muscle strength. CCL2 modulates neutrophils and CD4^+^ T helper cell recruitment to sustain SMW by impairing skeletal muscle formation and/or function. These data demonstrate important complex roles for CCL2–CCR2 signaling in regulating SMW.

### Significance of phenotypes in the 4T1 versus intramuscular injection models

Here, the 4T1 model showed reduced grip strength and fat mass and increased expression of SMW biomarkers, modeling cachexia. These data are consistent with studies demonstrating decreased muscle function in the 4T1 model (Ahmadabadi et al., 2020; Molanouri Shamsi et al., 2017; Wang et al., 2018). Although published studies have shown decreased skeletal muscle mass in the 4T1 model (Ahmadabadi et al., 2020; Molanouri Shamsi et al., 2017; Wang et al., 2018), we found minor changes in lean mass compared to that in healthy mice. As we observed decreased myofiber size in 4T1 tumor-bearing mice, changes in skeletal muscle mass could have been masked by other tissue types in the EchoMRI lean mass assessments.

Given the partial phenotypes with CCL2 knockdown, we considered whether CCL2 levels in the 4T1 model were sufficient to functionally contribute to SMW. CCL2 expression levels in cultured 4T1 cells vary among studies (Fang et al., 2012; Rego et al., 2014). Technical and biological factors could have affected results, including differing sensitivities among ELISA kits and protocols (Furuya et al., 2019; Kinn Rød et al., 2017), and differences in cell density, which affects cytokine expression (Sharif and Wellstein, 2015; Sukho et al., 2017; Vaughan-Jackson et al., 2022). *In vivo*, the serum levels of CCL2 are elevated after establishment of 4T1 tumors (Yoshimura et al., 2013). Furthermore, subpopulations within the 4T1 cell line express particularly high levels of CCL2, corresponding to metastasis (Bonapace et al., 2014; Yoshimura et al., 2023). Thus, CCL2 expression in 4T1 carcinoma cells may be associated with disease progression. CCL2 expression in breast cancer stroma (Imamura et al., 2021; Yoshimura et al., 2013) may also contribute to the overall levels of CCL2 levels observed in tissues and in serum. These studies indicate that the 4T1 model is suitable for examining the functions of CCL2. The partial effects of tumoral CCL2 knockdown on SMW indicate that other tumor-derived factors compensate for CCL2 deficiency, which could be identified through unbiased molecular profiling of tumor and muscle tissues.

Here, high levels of CCL2 adversely affected muscle cells and tissues. Notably, high levels of CCL2 inhibited grip strength *in vivo* and inhibited myoblast and myotube activity, increased AMPK, SMAD3 and JNK pathway signaling, and decreased activation of SRC *in vitro*. High levels of CCL2 could increase the levels of chemokine dimerization, which modulates ligand–receptor binding and directs downstream signaling (Blanchet et al., 2023). This would involve downregulation of signaling pathways associated with mitogenesis and differentiation, and enhanced signaling pathways associated with proteasomal degradation, apoptosis and metabolic dysregulation. Is a low level of CCL2 beneficial? A low level of CCL2 enhanced grip strength and expression of MYL *in vivo*, and increased myoblast proliferation *in vitro*. However, a low level of CCL2 also decreased MYH2 expression compared to that in control tissues, indicating that a low dose of CCL2 may have unforeseen effects on muscle. We conclude that a low level of CCL2 activates myoblasts and is associated with higher grip strength. We cannot draw conclusions on its potential benefits.

Here, high CCL2 treatment and tumoral CCL2 knockdown resulted in some complementary phenotypes, including changes in myoblast proliferation, myotube apoptosis, and expression of atrogin-1 and LC3B biomarkers *in vitro* and *in vivo*. Furthermore, *in vivo* CCL2 expression in muscle was negatively associated with myofiber size and alterations in MYH and MYL expression in the CCL2 delivery and 4T1 tumor models. Interestingly, although high CCL2 treatment increased MuRF1 expression in myotubes *in vitro*, *in vivo* CCL2 delivery and tumoral CCL2 knockdown *in vitro* did not affect MuRF1 expression. This could have been due to the tissue microenvironment versus that in tissue culture plates *in vitro*. We also observed non-complementary phenotypes between tumoral CCL2 knockdown and CCL2 delivery, notably *in vivo*. High-dose CCL2 but not the 4T1 tumors reduced overall MYH expression and the expression of fast-twitch and slow-twitch biomarkers, which were associated with decreased grip strength. In contrast, 4T1 tumors did not yield statistically significant differences in expression of MYH fast- and slow-twitch biomarkers compared to that in normal healthy mice. These data indicate that CCL2 intramuscular injection mimics some but not all features of a CCL2-expressing mammary tumor model, and that additional 4T1 tumor-derived factors could affect the expression of MYH and MYL in muscle. There were unexpected results with the intramuscular injection and 4T1 models. For one, tumoral CCL2 knockdown but not intramuscular injection affected MuRF1 expression. These differences could be due to co-expressing factors from 4T1 tumors that regulate its expression. These factors could act directly on muscle tissue or indirectly through the immune microenvironment to affect MuRF1 expression. MuRF1 expression could also depend on CCL2 concentrations; intramuscular doses of CCL2 may not reflect tumor-derived CCL2 levels in the muscle. In addition, CCL2 delivery significantly affected grip strength but not lean mass, although studies have reported a positive correlation between lean mass and muscle strength and an inverse correlation between fat mass and lean mass in healthy women (Ben Mansour et al., 2021; Chan et al., 2022). As CCR2 is expressed in neuronal cells (Komiya et al., 2020), high-dose CCL2 could have adversely affected muscle strength through these mechanisms without affecting muscle mass in the duration of experiments. The decreased muscle strength and fat mass associated with high-dose CCL2 are consistent with trends observed during cancer cachexia, as demonstrated in the 4T1 model here and in the Lewis lung carcinoma model (Raun et al., 2023). Conversely, CCL2 knockdown significantly affected lean mass but not grip strength. Increased muscle strength could have followed the increase in lean mass if the endpoint had been extended. Another possibility is that tumoral CCL2 knockdown was not sufficient to affect neuronal stimulation to muscle tissues to improve muscle function. Thus, intramuscular injection of CCL2 does not completely model the effects of tumor derived CCL2 on muscle. We note that CCL2 knockdown in the 4T1 model enhanced some muscle phenotypes over those in healthy mice, such as lean mass. However, the difference in lean mass between healthy and 4T1.CCL2si mice was 0.76%, which could be within the range of physiological variability. As tumor growth was not significantly affected in 4T1.CCL2si mice, they may exhibit physiological phenotypes not found in healthy mice.

### Significance of phenotypes in the 4T1 versus MDA-MB-231 models

Our studies demonstrated that the 4T1 and MDA-MB-231 models enhanced SMW associated with reduced myoblast proliferation, and increased myotube apoptosis and lactate production. CCL2 knockdown in both models reduced the expression of SMW biomarkers, demonstrating similarities between the models. Yet, there were also phenotypic differences. Treatment with 4T1-CM but not MDA-MB-231-CM increased CCR2 expression in myoblasts and reduced the number of myotube nuclei. These discrepancies could be due to differences in CCL2 levels between cell lines (Fang et al., 2012). MDA-MB-231 cells express factors such as IGF1 (Cullen et al., 1992; Lin et al., 2021), which could interfere with CCL2 during the myotube differentiation process. Interestingly, CCL2 knockdown in the MDA-MB-231 model (Fang et al., 2016) but not in the 4T1 model inhibited tumor growth. This could potentially be due to differences in the genetic background of mice. Here, 4T1 tumors were established in BALB/c mice, in which an increase in myeloid-derived suppressor cells and T regs and a decrease in cytotoxic T cells are important mechanisms for 4T1 tumor progression (Bosiljcic et al., 2019; Shen et al., 2018). T cells would be lacking in immunocompromised mice with MDA-MB-231 xenografts, and macrophages are important for mammary tumor growth in nude mice (Fang et al., 2016; Hembruff et al., 2010). The differences in the immune system could have affected tumor growth with CCL2 knockdown.

### Significance of CCL2–CCR2 signaling to muscle cells

We demonstrate that CCL2 regulates the activity of myoblasts and myotubes through CCR2-dependent mechanisms. Interestingly, CCL2 or 4T1-CM treatment enhanced CCR2 expression in myoblasts but not in myotubes. Pharmacological inhibition of CCR2 rescued the inhibitory effects of CCL2 in myoblasts and myotubes, indicating that CCR2 activity is important for CCL2 signaling in both cell states. Here, high-level CCL2 treatment increased JNK, AMPK and SMAD3 pathway signaling and reduced phosphorylation of components of mitogenic pathways to inhibit myoblast and myotube activity. These findings are consistent with studies demonstrating that these signaling pathways promote skeletal muscle atrophy in models of denervation, colon cancer and pancreatic cancer (Goodman et al., 2013; Mulder et al., 2020; Umezu et al., 2021; White et al., 2013). Additionally, CCL2-induced enhancement of SMAD3 and FOXO3 activity and decreased expression of PPARγ and PGCα are consistent with studies demonstrating opposing phenotypes for SMAD3/FOXO activity and PPARγ in muscle (Bollinger et al., 2014; Crossland et al., 2021; Goodman et al., 2013; Kupr and Handschin, 2015). Yet, JNK and SMAD3 signaling positively regulates the growth of new skeletal muscle during exercise or tissue injury (Ge et al., 2012; Lessard et al., 2018). AMPK agonists restored muscle cell activity impaired by TNFα and IFNγ and partly alleviated SMW in a transplant model of colon cancer, associated with increased oxidative metabolism (Hall et al., 2018). As JNK inhibition further decreased myoblast proliferation in CCL2-treated myoblasts, the relevance of JNK activity on myoblasts could be further clarified in the future using approaches such as CRISPR or siRNA knockdown. Overall, the role of JNK, SMAD3 and AMPK in skeletal muscle could depend on regulatory factors, physiological conditions and/or mouse models involved.

We observed complex effects of CCL2 on myotube formation. Notably, high concentrations of CCL2 resulted in lower myotube fusion index, whereas 4T1-CM treatment led to a higher fusion index. This higher index is due to a lower total number of nuclei from 4T1-CM treatment, which raised the proportion of myotube nuclei. The lower total number of nuclei may be due to increased death of myoblasts and/or differentiated myotubes, or decreased numbers of proliferating myoblasts. The decreased myotube formation with 4T1-CM treatment is consistent with studies showing that conditioned medium from ovarian, prostate and colon cancer cells inhibits myotube formation based on the myotube fusion index (Baccam et al., 2019; Marchildon et al., 2015). In one study, conditioned media from human breast cancer cell lines accelerated the differentiation of human muscle stem cells but reduced the expression of dystrophin and MYH in differentiated muscle cells associated with decreased AKT activity (Wang et al., 2023). The differences in myotube differentiation between studies could be partly due to the breast cancer cell lines used, primary muscle cells versus murine C2C12 cells used, and species-dependent effects on myotube differentiation. The adverse effects on differentiated muscle cells observed in published studies support an important role for breast cancer-derived factors in SMW.

We demonstrate an important role for CCL2–CCR2 signaling in metabolism. Although lactate is produced under normal activities such as exercise, increased lactate is associated with colon cancer-associated cachexia (Visavadiya et al., 2021; Wei et al., 2022). Here, CCL2 treatment or treatment with CCL2 derived from 4T1 cells elevated lactate production, which is associated with decreased survival, suggesting that this metabolic phenotype is associated with SMW. Interestingly, CCL2 treatment did not affect ATP production, a phenotype observed in some models of cancer cachexia (Constantinou et al., 2011; Tzika et al., 2013) but not in others (Julienne et al., 2012). ATP levels decreased due to mitochondrial dysfunction or were required for protein degradation during SMW (Constantinou et al., 2011; White et al., 2011). The increased lactate production could be part of broader metabolic changes regulated by CCL2 signaling, which could be identified through metabolomics and isotope-tracing studies. In contrast to CCL2 treatment, 4T1-CM decreased ATP production in myotube cultures, which is consistent with studies on colon cancer models (Constantinou et al., 2011; Tzika et al., 2013). Different soluble factors expressed in 4T1-CM may affect metabolism in myotube cultures, which could be identified through transcriptomics or proteomics screening.

Some differences in results were observed with siRNA and shRNA knockdown of CCL2. Although CCL2 siRNA and shRNA knockdown rescued myoblast proliferation and myotube activity, control and CCL2 shRNA-expressing cells both resulted in a higher myotube fusion index. Notably, 4T1 control cells stably expressing EGFP shRNAs did not reduce the number of myotube nuclei, in contrast to 4T1 control siRNA-transfected cells. Although the reasons are unclear, off-target effects have been reported with GFP knockdown, shRNA stable expression and lentiviral transduction (Klinghoffer et al., 2010; Mockenhaupt et al., 2015; Tschuch et al., 2008). Culturing conditions were different for myoblast proliferation assays, myotube formation assays and myotube activity assays. Off-target effects from one or more of these processes could have affected cellular differentiation.

### Significance of CCL2-mediated alterations in the immune microenvironment to SMW

Here, tumoral CCL2 knockdown altered the levels of neutrophils and CD4^+^ T helper cells in skeletal muscle, corresponding to increased lean mass and reduced expression of markers of muscle atrophy, suggesting a muscle regenerative role for neutrophils and T cells. This is supported by previous studies: CD4^+^ T cells enhance proliferation of myoblasts and support muscle regeneration (Ziemkiewicz et al., 2021), and neutrophils correlate with the activation of satellite cells after tissue injury (Koike et al., 2022). Although CCL2 regulates macrophage recruitment and activation in response to muscle injury (Wang and Zhou, 2022), tumoral CCL2 knockdown did not alter macrophage levels in muscle tissues. These data suggest that tumoral CCL2 modulates immune responses to skeletal muscle differently from CCL2-mediated muscle regeneration.

How might neutrophil and T cell recruitment be affected by tumoral knockdown of CCL2? Tumoral CCL2 knockdown could have upregulated cytokines that promote immune infiltration. Alternatively, CCL2 knockdown could have downregulated cytokines that inhibit their infiltration. CD4^+^ T cell recruitment is positively regulated by CCL5 and CCL21 and negatively regulated by IL35 (Zhang et al., 2020). Neutrophils are regulated by CXCL1 and CXCL2 (Carnevale et al., 2023). Expression of these cytokines could be affected by decreased CCL2 expression and signaling to endothelial cells, dendritic cells, T regs and macrophages in muscle (Hao et al., 2020). Moreover, CCL2 knockdown could have altered production of cytokines from tumor tissues that systemically affect muscle tissue. Investigating the role and mechanisms of immune cell recruitment in muscle could be performed in future studies.

### Conclusions

Targeting the CCL2–CCR2 pathway could partially benefit patients with breast cancer with SMW. CCR2 antagonists, tested in clinical trials for pancreatic cancer treatment (Fei et al., 2021; Wang et al., 2022), could be repurposed for patients with breast cancer. Considering the role of CCL2–CCR2 signaling in mediating muscle repair (Blanc et al., 2020; Ferrara et al., 2023), modulating the levels of CCL2 expression or activity rather than complete ablation is recommended. The functional contribution of tumor-derived CCL2–CCR2 signaling in SMW in the context of age and chemotherapy still needs investigation. Evaluating the role of CCL2 in SMW associated with breast cancer using multiple animal models may provide further insight in developing new therapeutic strategies to treat this condition in patients with breast cancer.

## MATERIALS AND METHODS

### Animal care and ethics statement

Female BALB/c mice that were 6-8 weeks old were purchased from Charles River Laboratories and maintained at the University of Kansas Medical Center in accordance with the Association for Assessment and Accreditation of Laboratory Animal Care. All animal procedures were conducted using an approved Institutional Animal Care and Use Committee protocol.

### Cell culture

C2C12, 4T1 and MDA-MB-231 cell lines were obtained from the American Tissue Culture Collection (ATCC; CRL-1772, CRL-2539 and HTB-26, respectively) and cultured in Dulbecco's modified Eagle medium (DMEM; Cytiva, SH30232.LS) with 10% fetal bovine serum (FBS; GenDEPOT, 50600-050) and streptomycin/penicillin. Cell lines stably expressing shRNAs were generated using approaches described previously (Hembruff et al., 2010). Cell lines were authenticated by ATCC using cell morphology checks and short tandem repeat profiling, and in the laboratory by flow cytometry for biomarker expression. Cells were tested for mycoplasma after thawing using the MycoAlert Plus Kit (Lonza, LT07-701) and were maintained for no longer than 4 months at a time.

### Generation of tumor conditioned medium

Approximately 100,000 cells/well in 24-well plates were incubated with 1 ml serum-free DMEM per well for 24 h. Conditioned media were collected and used in experiments where indicated, with or without 2% horse serum (Gibco, 16050-122) added.

### Reagents

The following reagents were used: recombinant murine CCL2 (R&D Systems, 479-JE), INCB3344 (Cayman Chemical, 28433), SP600125 (LC Laboratories, S-7929), SIS3 (Cayman Chemical, 15945) and BAY3827 (MedChem Express, HY-112083).

### Intramuscular injection

BALB/c female mice were anesthetized with 1-2% isoflurane and injected into the left and right quadricep muscle with 50 µl of 10 or 50 ng recombinant CCL2 or PBS vehicle control. Injections were performed twice a week for up to 4 weeks. Mice were then euthanized and tissues were harvested for analysis.

### Intra-tumoral injection of Ca-TAT/siRNA complexes

TAT peptides and siRNAs were synthesized and complexed as described previously (Fang et al., 2016). 100 μl containing approximately 100,000 4T1 cells was injected into the fourth and fifth inguinal mammary fat pads of female BALB/c mice. One week after transplantation, when tumors grew to approximately 0.25 cm in diameter, they were injected with 10 μg/100 μl of Ca-TAT/siRNA complexes at three different areas. Intra-tumoral injections were performed 3 days a week for up to 3 weeks, when control tumors reached approximately 1.5 cm in diameter, the maximum allowable size according to institutional guidelines. At endpoint, mice were euthanized and tissues were harvested for analysis.

### Grip strength measurements

The San Diego Instruments Grip Strength System was used to measure grip strength in mice. A digital force gauge, fitted with a grip grid for mice, records the maximal amount of force exerted for each mouse to grasp and hold the grid. Grip strength was measured by holding the mouse by the back of the neck in one hand and the tail with the other, allowing hindlimbs or all four limbs to attach to the grid, and pulling back gently. Four or five replicate measurements were obtained per mouse per time point and averaged. Grip strength was measured once a week following intramuscular or intra-tumoral injections.

### Body composition analysis

Body composition was measured using a magnetic resonance imager, the EchoMRI 1100 Analyzer (Houston, TX, USA). Individual mice were scanned once a week. The percentages of lean mass and fat mass were calculated from total body mass minus tumor mass.

### Blood serum collection and processing

Approximately 200 µl of blood was collected by cardiac puncture using procedures described previously (Parasuraman et al., 2010). Serum was extracted by centrifuging samples at 1000 ***g*** for 10 min at 4°C.

### Histology and immunohistochemistry

Tissues were fixed in 10% neutral formalin buffer (NBF), processed into paraffin, sectioned at 5 μm thickness using a Leica RM2125 RTS microtome (NCI, Inc., 1492125RTS1), dewaxed and stained with Hematoxylin and Eosin (H&E) as described previously (Fang et al., 2016). For immunostaining, antigen retrieval was performed by heating slides for 5 min at 100°C in a pressure cooker in 1 M urea (for CCL2, MYH, MYL, MYH2 and MYH4), Tris-EDTA at pH 9.0 (for MuRF1 and atrogin-1) or 10 mM sodium citrate at pH 6.8 (for LC3B). Slides were washed in PBS, incubated in PBS containing 10% methanol and 10% H_2_O_2_ for 10 min to quench endogenous peroxidases. For atrogin-1 and MuRF1, sections were blocked in PBS containing 3% FBS. For expression of all other proteins, sections were blocked in PBS containing 3.5% mouse FAB fragment (VWR, 102649-994). Slides were incubated in PBS containing 3% FBS and antibodies described in Table S9 at a 1:100 dilution. For CCL2, atrogin-1, MurF1, LCB3B, MYH or MYL immunostaining, sections were incubated with appropriate secondary biotinylated antibodies (1:1000) bound to streptavidin peroxidase (Vector Laboratories, PK6200). Reactions were catalyzed with 3,3ʹ-diaminobenzidine substrate (DAB). DAB-stained sections were counterstained with Mayer's Hematoxylin and mounted with Cytoseal (Richard-Allan Scientific/PHC Holdings, 8310-4). For MYH2 and MYH4 immunostaining, sections were incubated with anti-mouse-Dylight 488 (1:1000; Immunoreagents, VWR, 10150-194), counterstained with DAPI and mounted with PBS containing 50% glycerol (PBS:glycerol). Four fields per section were captured using the FL-Auto EVOS Imaging System (Thermo Fisher Scientific, AMAFD1000). Slides were sectioned and stained and analyzed in three to four different batches at a time for *in vivo* models by two different staff members.

### ImageJ quantification of biomarker expression and myofiber size

Biomarker expression in tissues or cells was measured as described previously (Yao et al., 2016). Briefly, images were imported into Adobe Photoshop. Images were normalized to white background using Auto Contrast and Auto Color. Using the selection tool, DAB staining or fluorochrome staining was highlighted, copied and saved as a separate file. The images were opened in ImageJ, converted to 8-bit images and analyzed for particle size. Positive staining was normalized to Hematoxylin or DAPI staining, as shown in graphs.

Cross-sectional area and minimum Feret diameter of H&E-stained myofibers was measured using methods adapted from previous studies (Akkad et al., 2014; Liu et al., 2013). Four images per section were captured at 20× magnification using the EVOS FL-Auto Imaging System. In ImageJ, individual muscle fibers were outlined using the freeform tool and the areas and Feret's diameters were measured.

### Flow cytometry

For immunophenotyping, 0.2 g muscle tissue was minced with fine surgical scissors and digested in DMEM:F12 (diluted 50/50; Cytiva, SH30243.LS and SH30010.04) containing 0.5 mg/ml collagenase (Sigma-Aldrich, 10103586001), 0.24 mg/ml hyaluronidase (Sigma-Aldrich, H3884), 20 mg/ml bovine serum albumin (Sigma-Aldrich, 2930) and 1× antibiotic/antimycotic Solution (GenDepot, CA002-010) for 1 h at 37°C with shaking. Samples were homogenized with a tissue tearer and quenched in Hanks’ Balanced Saline Solution (HBSS) with 2% FBS. Cells were pelleted by centrifugation, passed through 70 μm filters and washed with HBSS containing 2% FBS. Samples were incubated with UV live/dead stain (Invitrogen, L23105) and then with the antibodies described at a 1:100 dilution (Table S8) for 30 min on ice. Cells were washed with PBS, fixed in 0.5% paraformaldehyde and washed with 1× wash buffer (Biolegend, 420201) before analysis. For analysis of C2C12 cells, 500,000 cells were detached with accutase (Thermo Fisher Scientific, SCR005), incubated in PBS with 1:100 anti-CCR2-FITC (BioLegend, 150607) for 30 min, or with 10 μg/ml propidium iodide (Invitrogen, P3566) and anti-annexin-V (1:100, Biolegend, 640945) for 15 min at room temperature. All samples were analyzed using the Cytek Aurora Flow Cytometer. Samples were normalized to their respective unstained controls.

### *In vitro* siRNA transfection

Approximately 50,000 cells/well in 24-well plates were transfected in 500 µl Opti-MEM (Thermo Fisher Scientific, 11-058-021) with 2 µg siRNA specific to control EGFP (Dharmacon, CTM-442666) or murine CCL2 (Dharmacon, CTM-442667) and 8 µl Lipofectamine 2000 (Invitrogen, 11668019). Cells were washed with PBS and recovered in DMEM containing 10% FBS with antibiotics.

### Assessment of myotube fusion index

Approximately 8000 myoblasts were seeded onto 96-well plates for 24 h and incubated in DMEM containing 2% horse serum for 5 days. Cells were assessed for myotube fusion index using approaches described previously (Hinkle et al., 2021; Noë et al., 2022). Briefly, cells were fixed in 10% NBF, permeabilized with methanol for 10 min at −20°C, blocked in PBS containing 3% FBS for 1 h and incubated with anti-myosin heavy chain (MYH; 1:100, Santa Cruz Biotechnology, sc-32732) for 24 h. Cells were washed in PBS three times, incubated with Alexa Fluor 568-conjugated donkey anti-mouse IgG (1:500, Invitrogen/Thermo Fisher Scientific, A10037) for 1 h, washed in PBS and counterstained with DAPI. Cells were mounted with PBS:glycerol. Four images per well were captured at 10× magnification using the FL-Auto EVOS Imaging system. Myotubes were defined by MYH-positive cell structures containing two or more nuclei. The number of myotube nuclei and total number of nuclei were measured using ImageJ. The myotube fusion index was calculated by dividing the number of myotube nuclei by the total number of nuclei.

### Immunofluorescence

Cells (10,000/well) seeded in 96-well plates were incubated in serum-free DMEM, DMEM containing 2% horse serum with or without recombinant protein or inhibitors, or tumor conditioned medium with 2% horse serum for 24 h. Cells were fixed in 10% NBF for 24 h, permeabilized with methanol for 10 min at −20°C, blocked in PBS containing 3% FBS for 1 h and immunostained (1:100) for PCNA, cleaved caspase-3, SMAD3 or FOXO3 (listed in Table S10) in blocking buffer for 24 h at 4°C. PCNA was detected using an Alexa Fluor 647-conjugated donkey anti-mouse secondary antibody (1:500, Invitrogen, A-31571). Cleaved caspase-3, SMAD3 and FOXO3 were detected using Alexa Fluor 647-conjugated donkey anti-rabbit (1:1000, Invitrogen, A-31573), Alexa Fluor 647-conjugated donkey anti-mouse (1:500, Invitrogen, A-31571) and Alexa Fluor 488-conjugated donkey anti-rabbit (1:500, Jackson Immunoresearch, 111-545-144) secondary antibodies, respectively. Samples were counterstained with DAPI and mounted with PBS:glycerol. Images (four fields per well) were captured at 10× magnification using the FL-Auto EVOS Imaging System.

### Cell viability assay

Approximately 8000 cells/well in 96-well plates were incubated in serum-free DMEM with or without inhibitors for 24 h. Cells were incubated with MTS reagent (Promega, PAG3580) according to the manufacturer's instructions. Absorbance was read at 490 nm using a Tecan Infinite M Plex plate reader.

### Lactate and ATP assays

Approximately 8000 cells/well in 96-well plates were treated for 24 h with 100 µl DMEM containing 2% horse serum with or without treatments. Based on previous studies (Galle et al., 2022; Zocchi et al., 2023), the Promega Lactate-Glo assay (Promega, J5021) was used to measure lactate production. Intracellular levels were measured according to the manufacturer's protocol. Intracellular ATP levels were analyzed using the Promega Cell Titer-Glo 2.0 Assay (Promega, G9242) according to the manufacturer's protocol. Luciferase activity was measured using the Tecan Infinite M Plex plate reader.

### Immunoblot

Approximately 500,000 cells/well from six-well plates were lysed with RIPA buffer containing 1× PBS, 0.5% sodium deoxycholate, 1% Nonidet P-40 and 0.1% SDS with protease and phosphatase inhibitors (Sigma-Aldrich, P8340). 30 µg protein was resolved in 10-15% SDS-PAGE gels and transferred to nitrocellulose membranes. Membranes were blocked in 10 ml PBS containing 0.05% Tween 20 and 3% dry milk (PBS-T) for 1 h, and incubated in 5 ml blocking buffer containing the primary antibodies listed in Table S10 at 1:1000 dilution for 24 h. Membranes were washed with PBS-T three times for 15 min each and incubated with 7 ml of the appropriate secondary antibodies (1:1000) conjugated to horse radish peroxidase (goat anti-rabbit-HRP, ImmunoReagents, GTXRb-003-DHRPX; goat anti-mouse-HRP, ImmunoReagents, GTXMu-003-DHRPX) for 1 h in blocking buffer. Blots were washed with PBS-T three times for 30 min each, developed with West Q Chemiluminescent Substrate (GenDEPOT, W3651-048) and imaged using a BioAnalytik Jena Imaging System (UVP ChemStudio 815, VWR, SP1126) with Vision Works software. Densitometry analysis was performed using ImageJ as described previously (Alonso Villela et al., 2020). Blots were stripped in Thermo Scientific Restore PLUS Western Blot Stripping Buffer (VWR, PI46430) for 15 min at room temperature, washed in PBS-T three times, and blocked for 1 h before being reblotted.

### ELISA

Conditioned media or blood sera were assayed by ELISA for murine CCL2 levels according to the manufacturer's instructions (BioLegend, 432704). Reactions were catalyzed using 1-Step Ultra Tetramethylbenzidine Substrate (Pierce, PI34028) and stopped with 2 N HCl. Absorbance was read at 450 nm using the Tecan Infinite M Plex plate reader.

### Strategies to reduce bias and statistical Analysis

Sample sizes were determined by power analysis using PS Software (v3.1, Vanderbilt University, Nashville, TN, USA). Preliminary data on lean mass were used to measure the effect size (Cohen's *d*=1.2) of 4T1.CCL2si versus control groups. Assuming normal distribution of data, the required sample size for determining the effect size at alpha 0.05/2=0.025 significance level is 13/group. This provides 80% power to detect differences in lean mass between groups. To reduce bias during *in vivo* studies, the treatment order was randomized with numbered mice. Batches of mice (*n*=3-4/group) were injected at different intervals by different individuals. Data collection was masked. *In vitro* experiments were plated in triplicate and performed at least three times. Statistical analysis was performed using GraphPad software v9.0. Two groups with Gaussian distribution were analyzed using the two-tailed unpaired *t*-test. For more than two groups with Gaussian distribution, statistical analysis was performed using one-way ANOVA with Bonferroni's post hoc comparison. Percentage groups of more than two were analyzed using Kruskal–Wallis test with Wilcoxon rank sum test for pairs of groups. Significance was determined by *P*<0.05.

## Supplementary Material

10.1242/dmm.050398_sup1 Supplementary information
