# Supplementary material for: CCL2 signaling promotes skeletal muscle wasting in non-tumor and breast tumor models
Source: Dis Model Mech. 2024 Sep 9;17(8):dmm050398. doi: 10.1242/dmm.050398 (PMC11413935; doi:10.1242/dmm.050398)
Supplement: Supplementary information [file dmm-17-050398-s1.pdf]

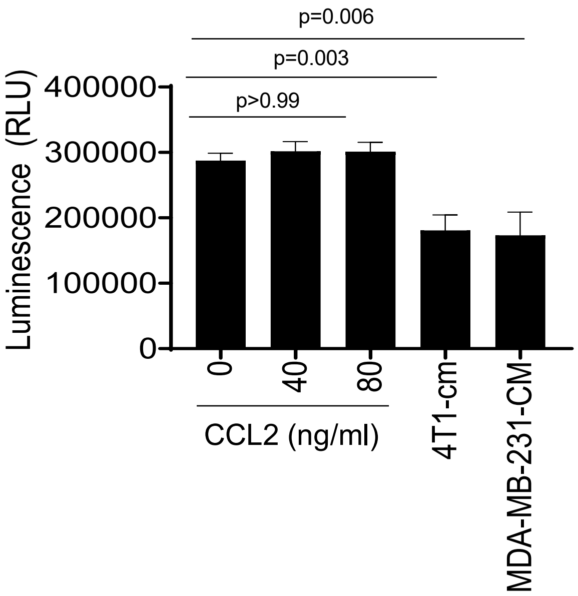

Fig. S1. 4T1 conditioned medium but not CCL2 treatment of C2C12 myotube cultures reduces ATP production. C2C12 myotubes were treated with CCL2 (40 or 80 ng/ml) or 4T1 or MDA-MB-231 tumor conditioned medium (CM) for 24 hours and analyzed for ATP production by biochemical assay. Experiments were performed 3 times with triplicate samples, n=9/group. Statistical analysis was performed using One Way ANOVA with Bonferroni post-hoc analysis. Significance was defined by $p<0.05$. Relevant post-hoc comparisons are indicated by line. Mean±SEM are shown.

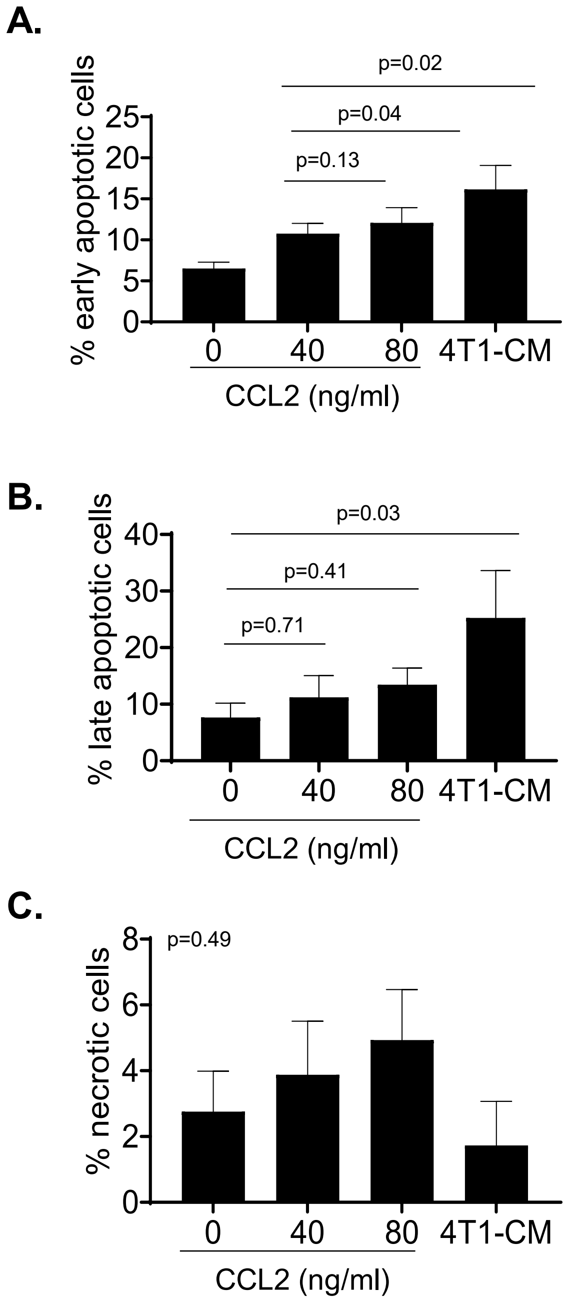

Fig. S2. CCL2 at high concentrations enhance apoptosis in myotube cultures by annexin V/ Propidium iodide staining. Myotube cultures were treated with CCL2 or 4T1 conditioned medium for 24 hours and analyzed for changes in annexin-V/Propidium iodide staining (PI) by flow cytometry. **A**. early apoptotic cells were identified by annexin-V+/PI- cells **B.** Late apoptosis were identified by annexin-V+/PI+ cells**. C.** Necrotic cells were identified by annexin-V-/PI+ cells. Experiments were performed 5 times, n=5/group. Statistical analysis was performed using One Way ANOVA with Bonferroni post-hoc analysis. Significance was defined by $p<0.05$. Relevant post-hoc comparisons are indicated by line. ANOVA p-value >0.05 is shown in the lefthand corner of C. Mean$\pm$SEM are shown.

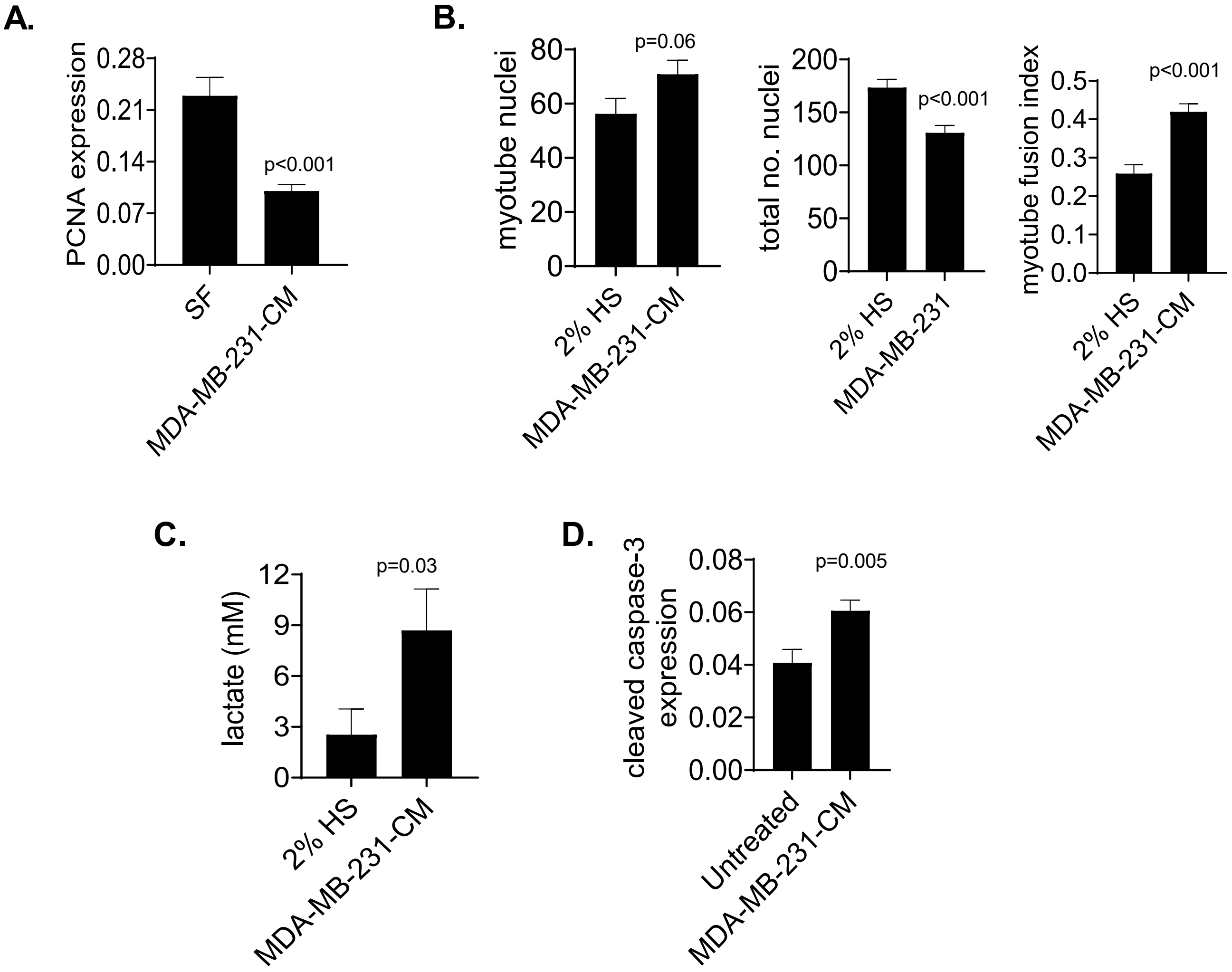

Fig. S3. Conditioned medium from MDA-MB-231 breast cancer cells inhibits the functions of C2C12 cells. A-B.C2C12 myoblasts were treated with control DMEM serum free (SF) medium, DMEM/2% horse serum (HS), or MDA-MB-231 conditioned medium with 2% HS and analyzed for cell proliferation by PCNA immunostaining (A) or myotube induction (B). **C-D.** Myotube cultures were treated with SF DMEM, DMEM/2% HS or MDA-MB-231-CM with 2% HS and analyzed for changes in intracellular lactate production by biochemical assay. SF below the limit of detection (C). Apoptosis was determined by cleaved caspase-3 immunostaining (D). Biomarker expression was quantified by Image J. For A-D, samples were plated in triplicate; experiments were performed 3 times (n=9/group).Statistical analysis was performed using Two Tailed Test. Statistical significance was defined by $p<0.05$. Mean±SEM are shown.

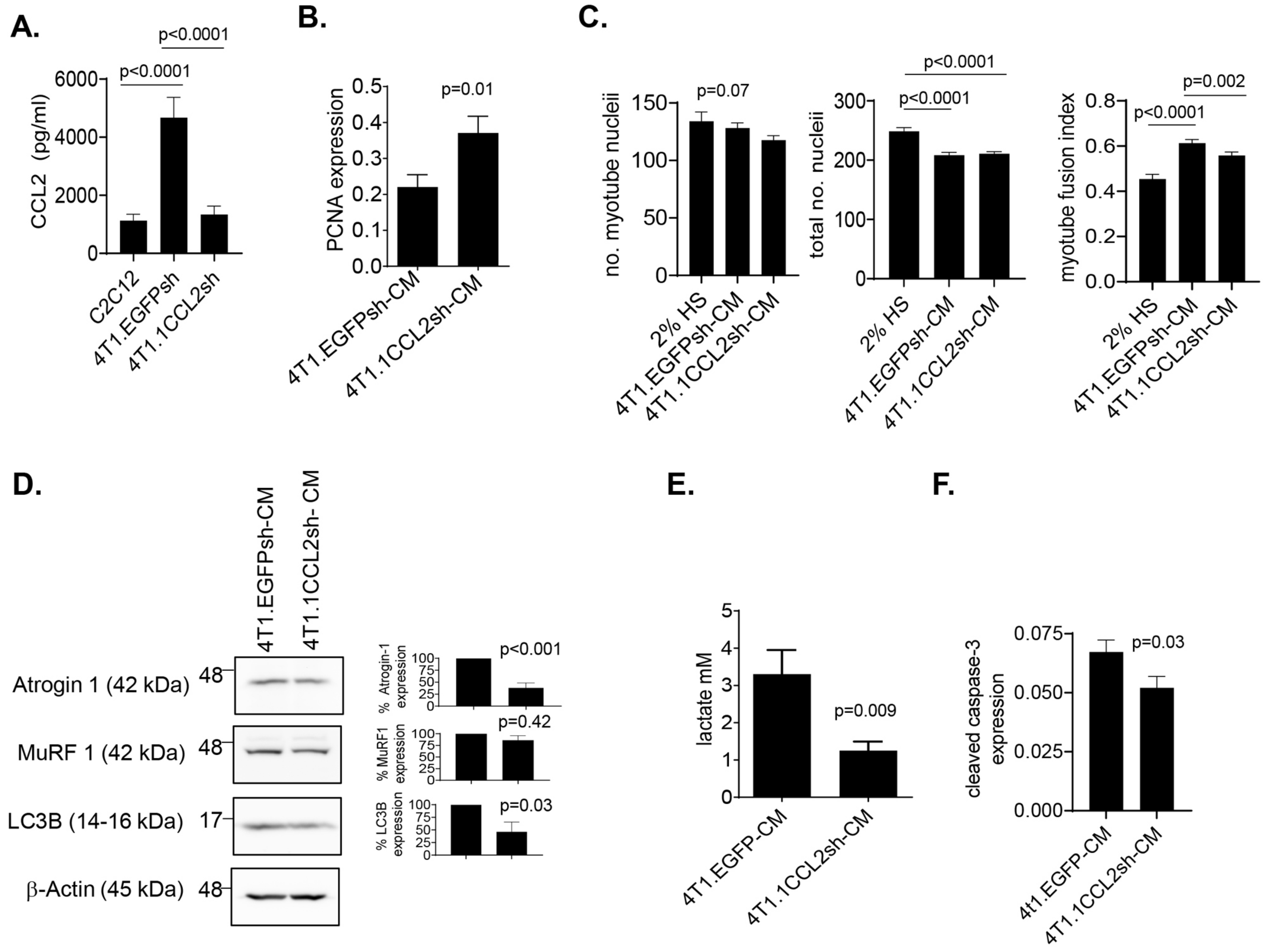

Fig. S4. Stable shRNA expression in 4T1 cells rescues activity of C2C12 myoblast and myotube activity. **A.** C2C12 myoblasts or 4T1 cells stably expressing control EGFP or CCL2 shRNAs were analyzed by ELISA by CCL2. **B-C.** C2C12 myoblasts were treated with DMEM serum free (SF) medium, DMEM/2% horse serum (HS) or 4T1 conditioned medium (CM) and analyzed for cell proliferation by PCNA immunostaining (B) or myotube induction (C). **D-E.** C2C12 myotubes were treated with DMEM/2% HS or 4T1-CM with 2% HS and analyzed for expression of the indicated proteins by immunoblot. Densitometry was performed on immunoblots. Samples were normalized to actin and expressed as a percentage relative to 4T1.EGFP-CM (D). Intracellular lactate levels were measured by biochemical assay (E). Apoptosis was determined by immunostaining for cleaved caspase-3 expression (F). Biomarker expression was quantified by Image J. For A-C, E-F, samples were plated in triplicate; experiments were performed 3 times (n=9/group). For C, experiments were repeated 4 times (n=4/ group). Statistical analysis was analyzed using One Way ANOVA with Bonferroni post hoc comparison (A,C), Wilcoxon Rank Sum Test (D) or Two tailed-test (B, E-F). Statistical significance was defined by p<0.05. For ANOVA p>0.05 , this p-value is shown in the lefthand corner of the graph. For ANOVA p<0.05, relevant post-hoc comparisons are indicated by line. Mean±SEM are shown.

Disease Models & Mechanisms • Supplementary information

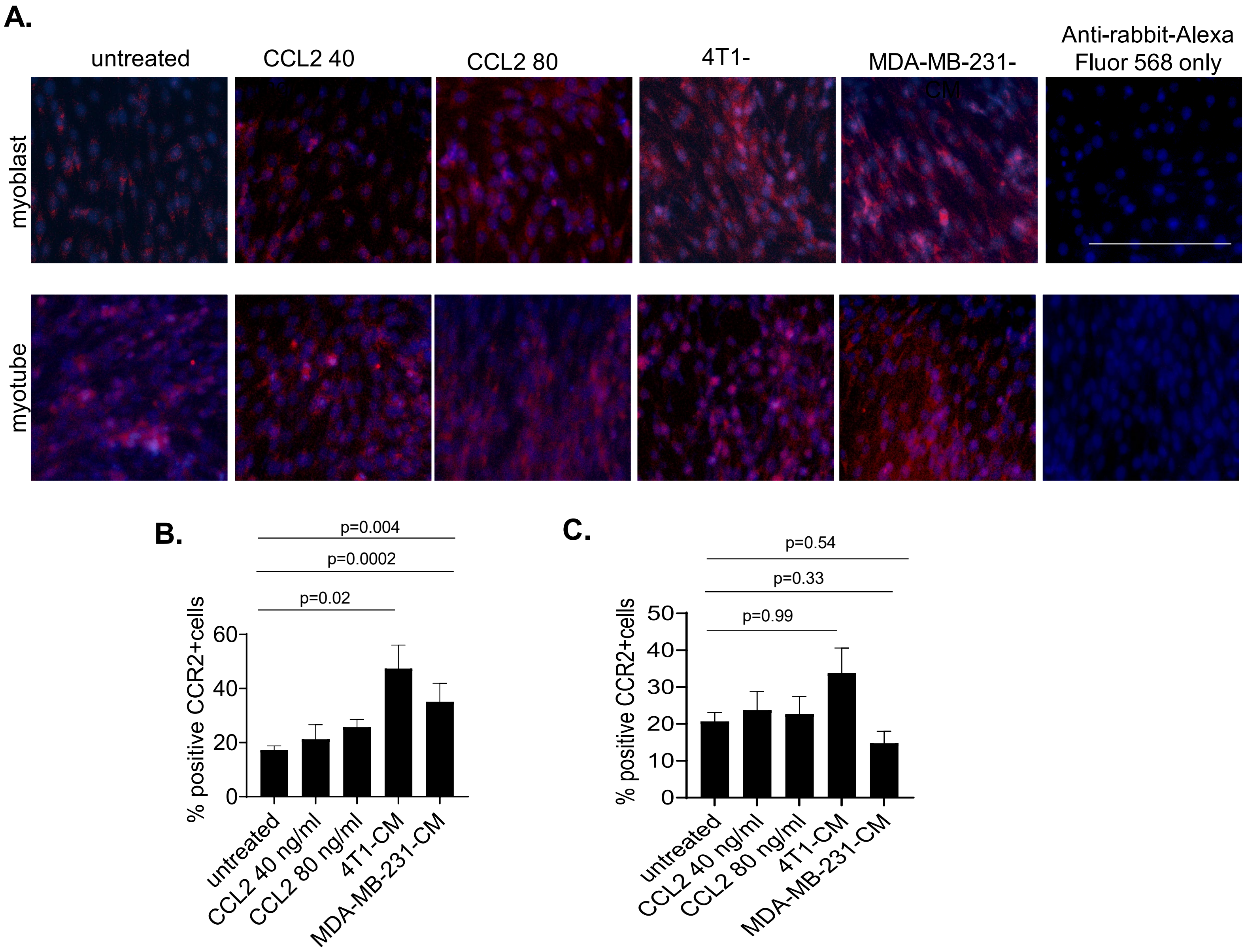

Fig. S5. Treatment of C2C12 cells with high concentrations of CCL2 or tumor conditioned medium enhances CCR2 expression in myoblasts. **A.** C2C12 myoblasts or myotube cultures were treated with/without 40 or 80 ng/ml CCL2 or 4T1 conditioned medium (4T1-CM) for 24 hours and analyzed for CCR2 expression by immunofluorescence staining. Scale bar=200 microns. **B.** Myoblasts or **C**. myotubes were treated with CCL2 or conditioned medium (CM) from 4T1 or MDA-MB-231 cells and analyzed for CCR2 expression by flow cytometry. For A, samples were plated in triplicate; experiments were performed 3 times (n=9/group). For B-C, experiments were repeated 5 times (n=5/group). Statistical analysis was analyzed using One Way ANOVA with Bonferroni post hoc comparison. Statistical significance was defined by $p<0.05$. Relevant post-hoc comparisons with $p<0.05$ are indicated by line. Mean$\pm$SEM are shown.

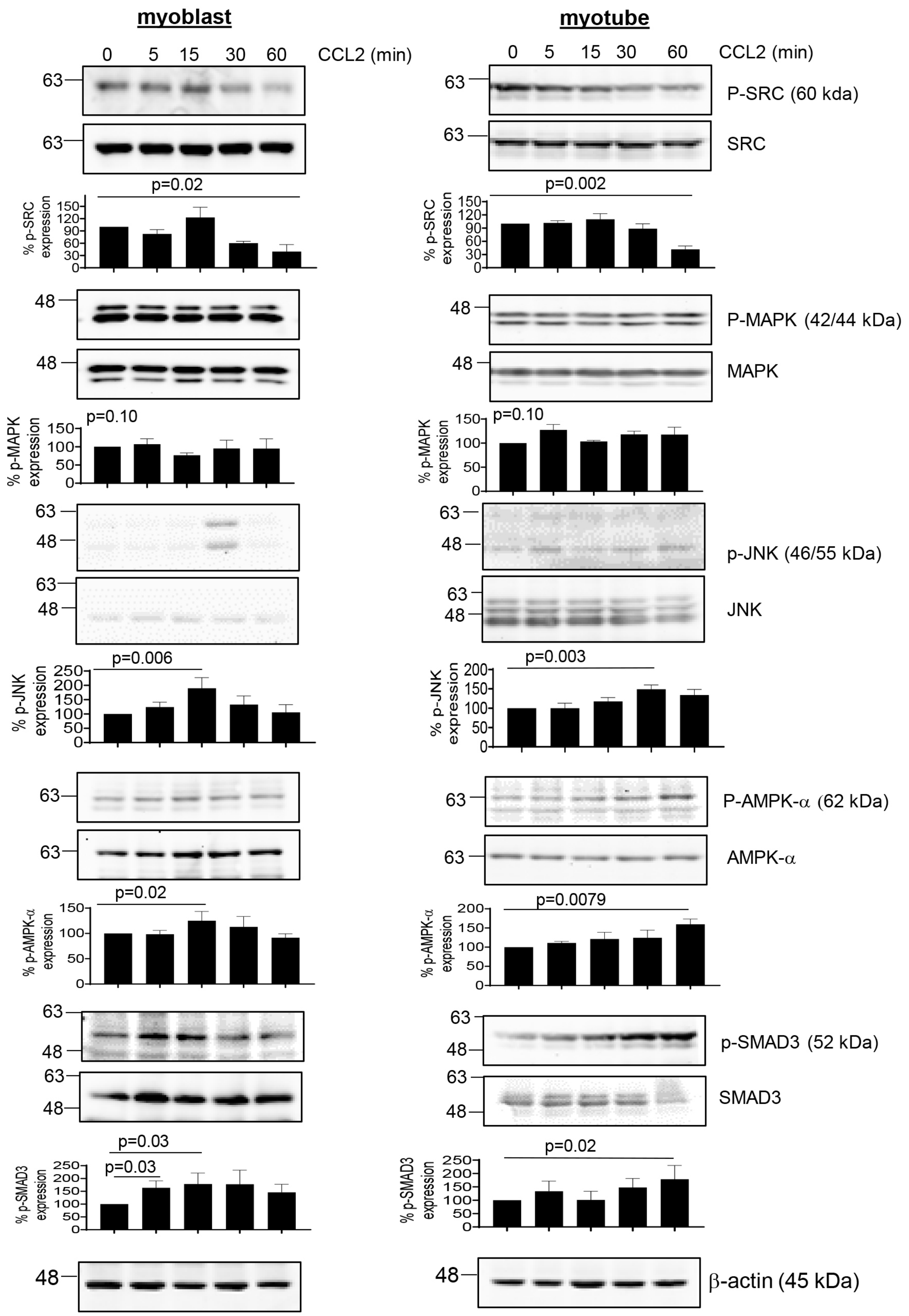

Fig. S6. CCL2 enhances JNK,SMAD3 and AMPK phosphorylation in C2C12 cells. C2C12 myoblasts and myotubes were treated with 80 ng/ml CCL2 for up to 1 hour and analyzed for expression of the indicated proteins by immunoblot. Experiments were performed 4-5 times, n=4-5/group. Statistical analysis was performed using Kruskall Wallis Test with Wilcox Rank Sum post-hoc analysis. Significance was defined by p<0.05. For Kruskal Wallis p-values >0.05, this p-value is shown in the lefthand corner of each graph. For Kruskal Wallis p<0.05, relevant post-hoc comparisons are indicated by line. Mean+SEM are shown.

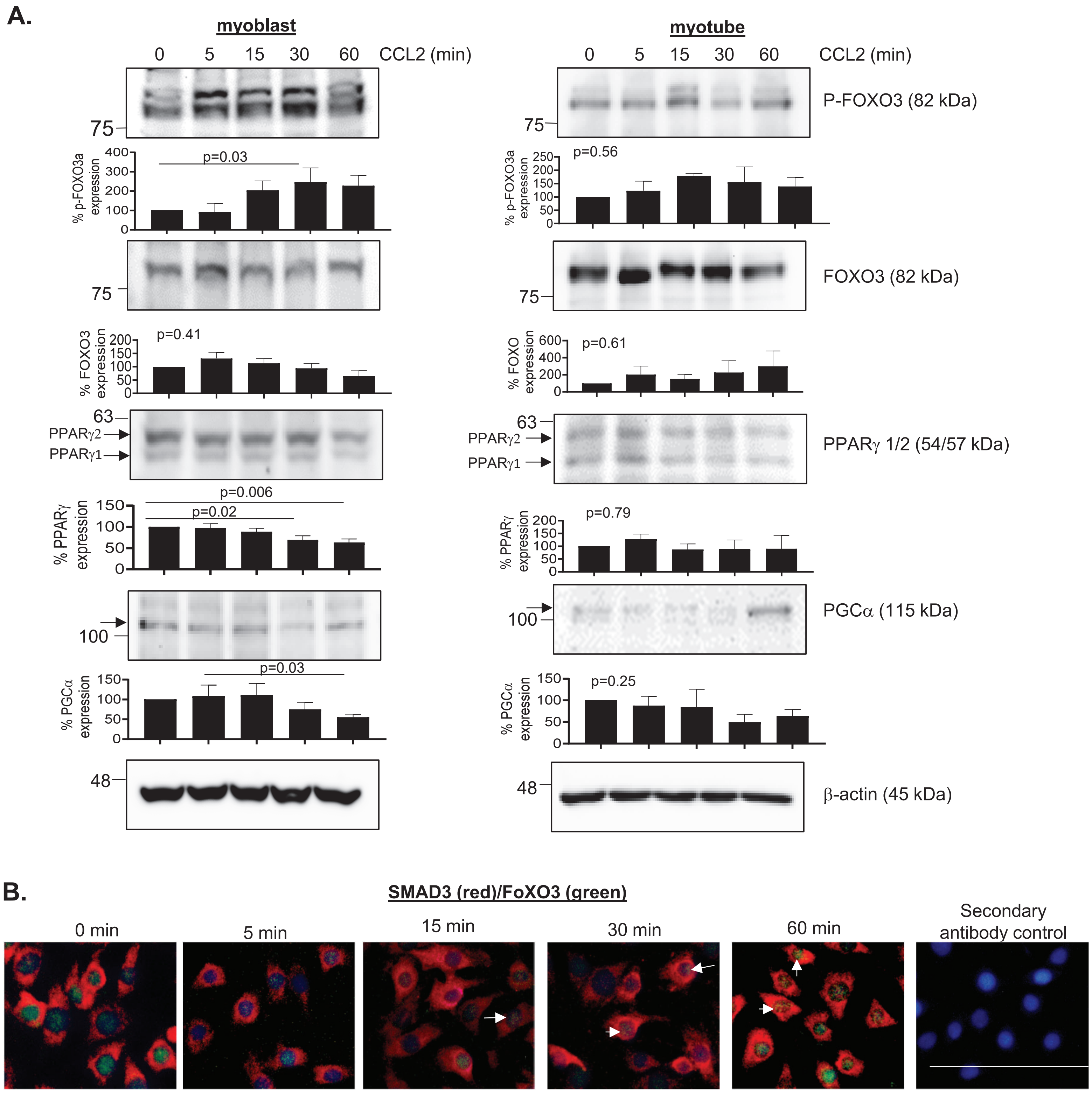

Fig. S7. CCL2 enhances expression and activity of FOXO3 and downregulates PPARγ and PCGα expression in myoblasts. A. C2C12 myoblasts and myotubes were treated with 80 ng/ml CCL2 for up to 60 minutes and analyzed for expression of the indicated proteins by immunoblot. Densitometry was performed on immunoblots. Samples were normalized to actin and expressed as a percentage relative to untreated. Mean +SEM are shown. **B.** C2C12 myoblasts were treated with CCL2 and analyzed for localization of SMAD3 (red) and FOXO3 (green) by co-immunofluorescence staining. Representative images with DAPI counterstain are shown. Secondary antibody controls are overlaid images of Anti-mouse alexa-Fluor-647/Anti-rabbit-Alexa-Fluor-488/DAPI. Scale bar= 100 microns. White arrows point to overlapping expression in the nucleus. For A, experiments were performed 4-5 times, n=4-5/group. For B, samples were plated in triplicate; experiments were performed 3 times (n=9/group). Representative images are shown. Statistical analysis was performed using Kruskal Wallis Test with Wilcox Rank Sum post-hoc analysis. Significance was defined by p<0.05. For Kruskal Wallis p->0.05, this p-value is shown in the lefthand corner of each graph. For Kruskal Wallis p<0.05, relevant post-hoc comparisons are indicated by line. Mean+SEM are shown.

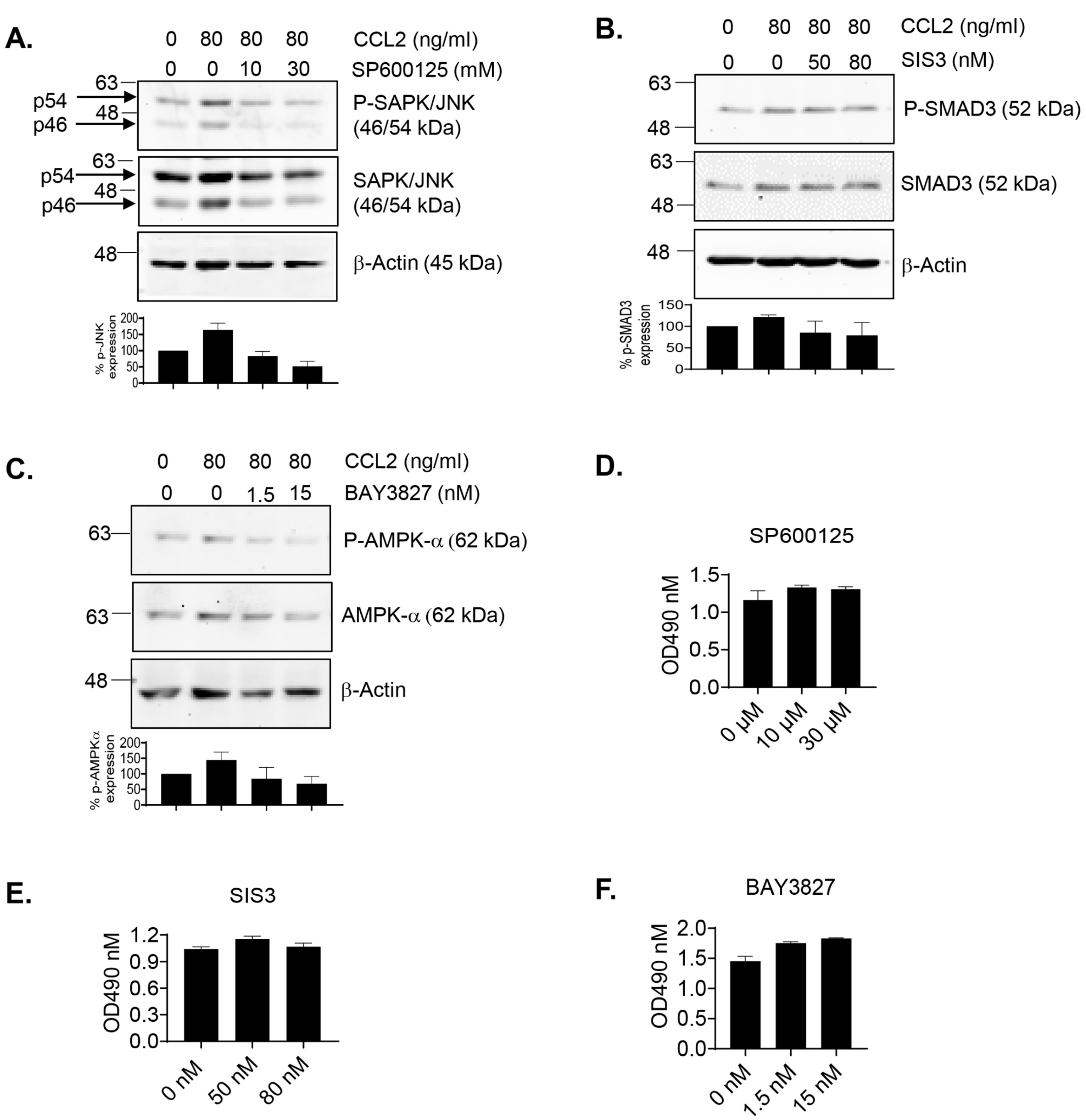

Fig. S8. Increasing concentrations of JNK, AMPK and SMAD3 pharmacologic inhibition in C2C212 cells decrease kinase phosphorylation and increase cell viability. A-C. C2C12 myoblasts were treated with: SP60125 (A), SIS3 (B) or BAY3827 (C) and analyzed for expression of the indicated proteins by immunoblot. Densitometry was performed on immunoblots. Samples were normalized to actin and expressed as a percentage relative to untreated. **D-F.** C2C12 myoblasts were treated with 80 ng/ml CCL2 in the presence or absence of SP600125 (D), SIS3 (E) or BAY3827 (F) and analyzed for cell viability by MTS assay. For A-C, experiments were performed 3 times, n=3/group. For D-F, samples were plated in triplicate and performed 3 times (n=9/group). Mean±SEM are shown.

Disease Models & Mechanisms • Supplementary information

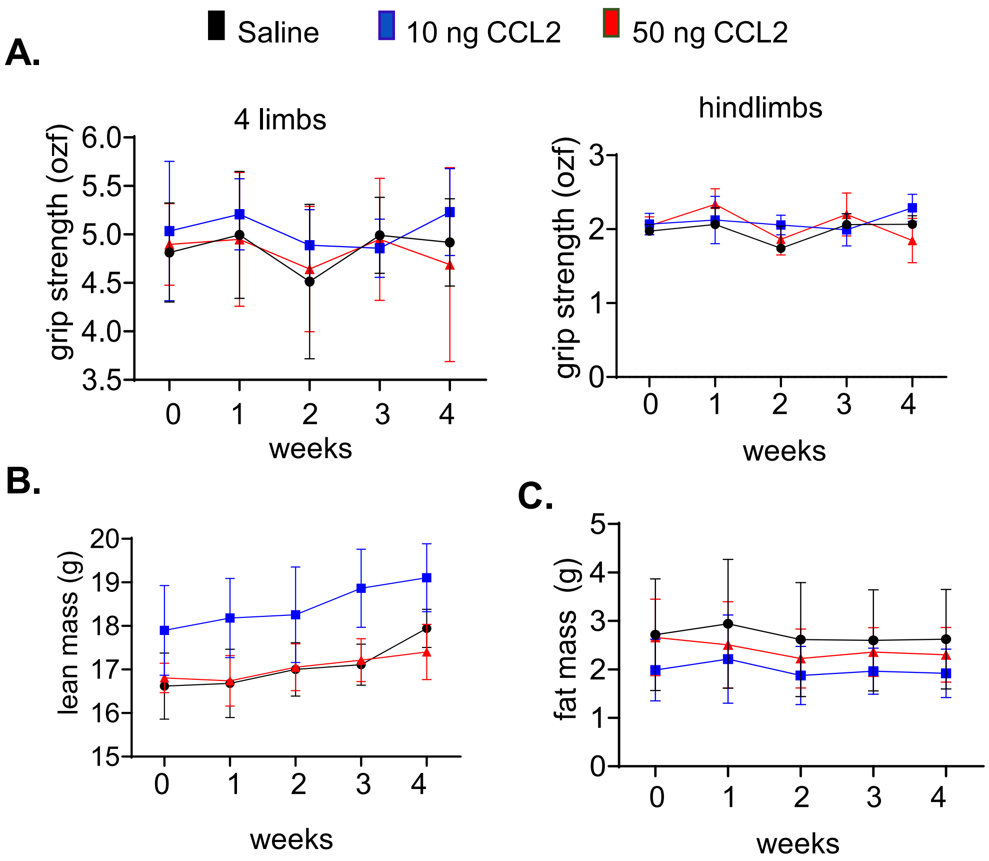

Fig. S9. Non-normalized grip strength and body mass values for mice treated with recombinant CCL2. **A.** Non-normalized grip strength values (ounce-force; ozf) for all 4 limbs (left graph) or hindlimbs (right graph), **B.** Non-normalized lean mass or **C.** Fat mass in female BALB/c mice treated with saline control, 10 ng or 50 ng recombinant CCL2 (n=9/group). Mean+STDEV are shown.

Disease Models & Mechanisms • Supplementary information

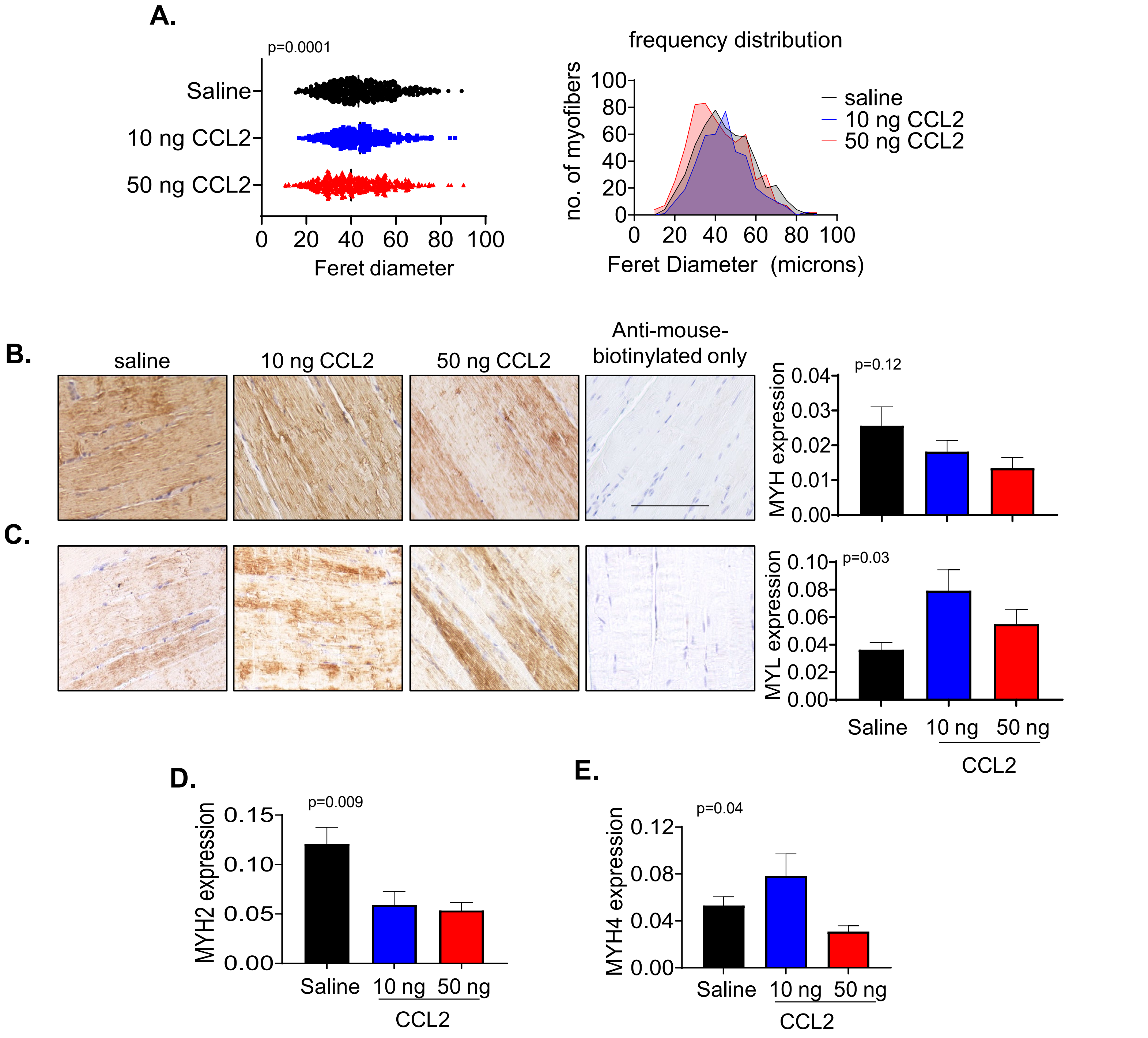

Fig. S10. CCL2 delivery alters myofiber size and expression of MYH and MYL proteins in skeletal muscle tissues. A. Feret diameter was measured on H&E stained sections of gastrocnemius myofibers. Left: scatter plot. Right: frequency distribution plot. 400-600 myofibers were measured per group. **B-F.** Gastrocnemius muscle in mice receiving saline, 10 ng or 50 ng CCL2 were immunostained for expression of MYH (B) MYL (C), MYH2 (D) or MYH4 (E). Longitudinal sections are shown. Expression was quantified by Image J. Statistical analysis was performed using One Way ANOVA with Bonferroni post-hoc comparison. Statistical significance was defined by $p<0.05$. Scale bar= 200 microns. For A-E, the ANOVA p-value is shown on the top lefthand corner of each graph. Mean±SEM are shown.

Disease Models & Mechanisms • Supplementary information

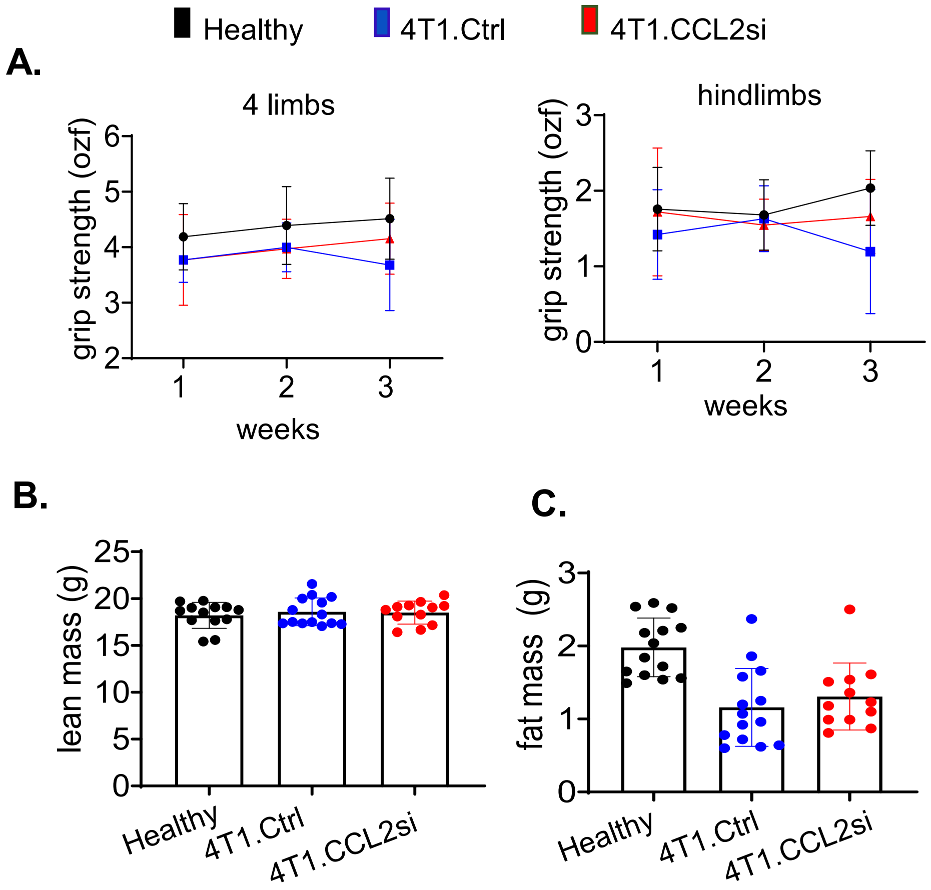

Fig. S11. Non-normalized grip strength and body masses with tumoral knockdown of CCL2. A. Non-normalized grip strength values (ounce-force; ozf) values of all 4 limbs (left graph) or hindlimbs (right graph) **B.** Non-normalized lean mass or **C.** Fat mass of 4T1 tumor bearing mice treated with Control (Ctrl), or CCL2 siRNAs (n=13/group). Healthy mice are a base-line control (n=14). Mean±STDEV are shown.

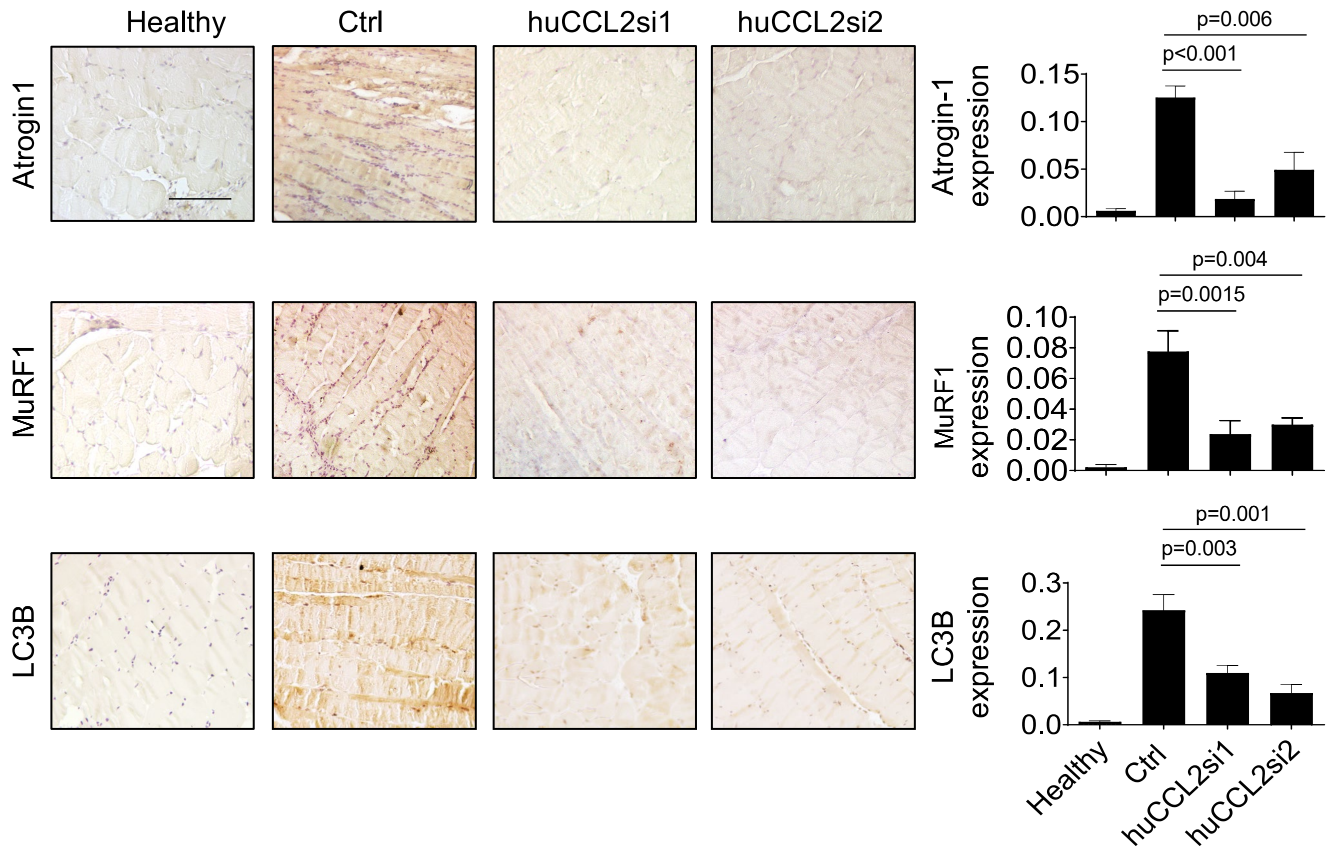

Fig. S12. CCL2 knockdown in MDA-MB-231 breast tumors xenografts reduces expression of Atrogin-1, MuRF1 and LC3B in skeletal muscle tissues. Quadricep muscle tissues were harvested form normal healthy mice or mice bearing MDA-MB-231 breast tumor xenografts treated with TAT cell penetrated peptides complexed to control (Ctrl) or CCL2 siRNAs (huCCL2si1, huCCL2si2). Muscle tissues were immunostained for expression of Atrogin-1, MuRF1 or LC3B. Expression was quantified by Image J (arbitrary units). N=4 per group. Scale bar=100 microns. Statistical analysis was performed using One Way ANOVA with Bonferroni post hoc comparison. Statistical significance was defined by $p<0.05$. Relevant post-hoc comparisons with $p<0.05$ are indicated by line. Mean±SEM are shown.

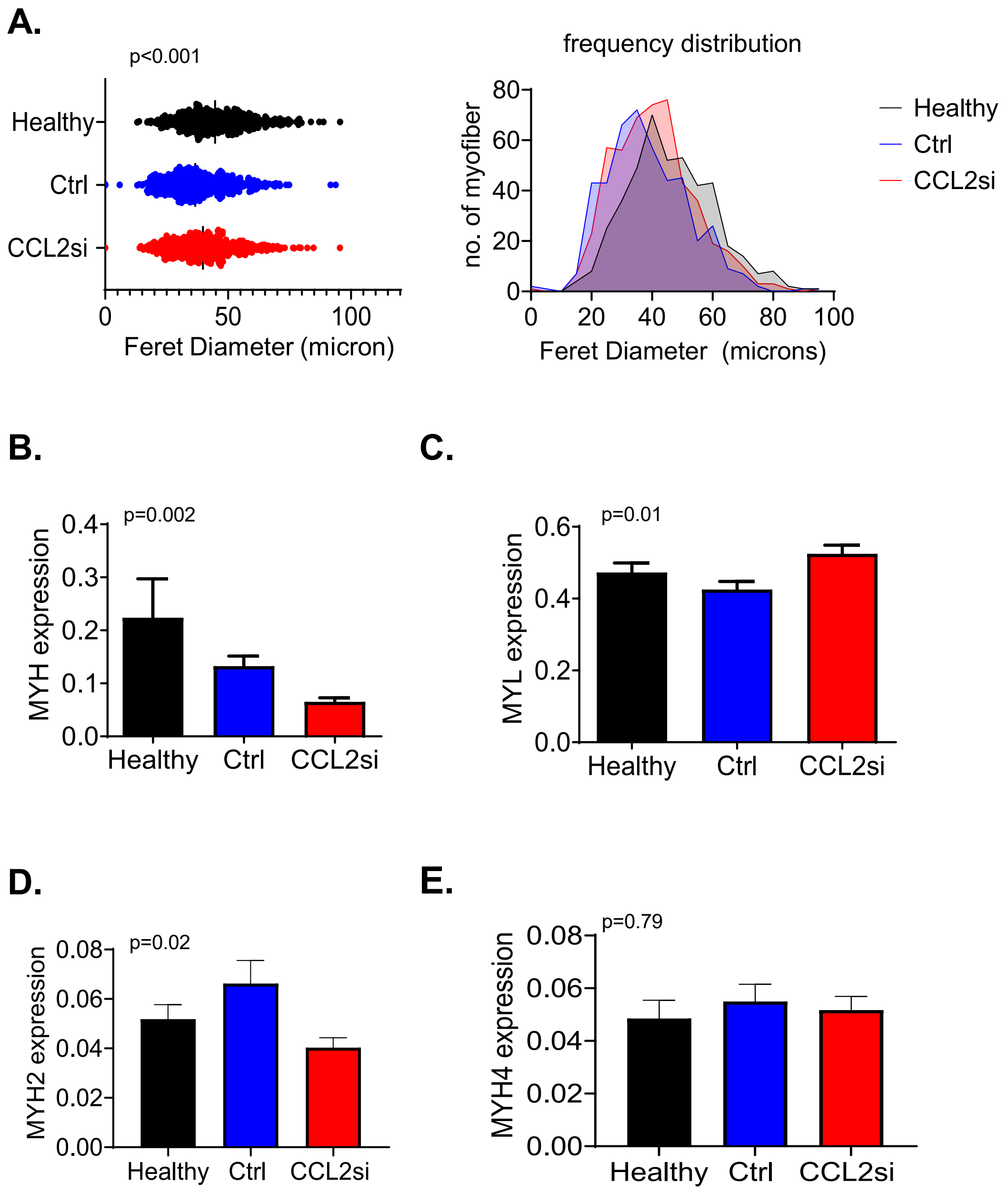

Fig. S13. Tumoral knockdown of CCL2 alters myofiber size and expression of MYH and MYL proteins in skeletal muscle tissues. A. H&E stained cross sections of gastrocnemius myofibers were measured for Feret Diameter. Left: scatter plot. Right: frequency distribution plot. 400-600 myofibers were measured per group **B-F.** Longitudinal sections of gastrocnemius muscle tissues in healthy or 4T1 tumor bearing mice with/without CCL2 knockdown were immunostained for expression of MYH (B), MYL (C), MYH2 (D) or MYH4 (E). Expression was quantified by Image J. Statistical analysis was performed using One Way ANOVA with Bonferroni post-hoc comparison. Statistical significance was defined by $p<0.05$. For A-E, the ANOVA p-value is shown on the top lefthand corner of each graph. Mean±SEM are shown.

Disease Models & Mechanisms • Supplementary information

Table S1. Summary of statistical comparisons in the intramuscular injection model for grip strength and body mass composition. Standardized mean differences (SMD) are shown. Statistical analysis was determined by One Way ANOVA with Bonferroni post-hoc analysis. Post-hoc p-values are shown.

		4 limb grip strength		hindlimb grip strength		lean mass composition		fat mass composition	
		SMD	p	SMD	p	SMD	p	SMD	p
Week 1	10 ng CCL2 vs. saline	0.78	0.16	-1.23	0.98	0.18	>0.9999	-0.0083	>0.99
	50 ng CCL2 vs. saline	-0.71	0.19	0.03	>0.99	0.95	0.09	-1.4	0.02
	50 ng CCL2 vs. 10 ng CCL2	-1.12	>0.99	0.5	0.9	0.95	0.21	-1.44	0.02
Week 4	10 ng CCL2 vs. saline	0.51	0.54	0.78	0.87	0.18	0.91	-0.039	>0.99
	50 ng CCL2 vs. saline	-0.75	0.21	-0.71	0.34	0.95	0.07	-0.47	0.81
	50 ng CCL2 vs. 10 ng CCL2	-1.39	0.027	-1.12	0.04	0.95	0.16	-1.76	0.93

Table S2. Summary statistical composition in the IM injection model for SMW biomarker expression and myofiber size analysis in muscle tissues. Standardized mean differences (SMD) are reported. Statistical analysis was determined by One Way ANOVA with Bonferroni post-hoc analysis. Post-hoc p-values are shown.

	CCL2 muscle		Atrogin-1		MurF1		LC3B		CSA		Feret Diameter	
	SMD	p	SMD	p	SMD	p	SMD	p	SMD	p	SMD	p
10 ng CCL2 vs. saline	0.11	>0.99	-0.21	0.9	-0.51	0.54	0.13	>0.99	-0.07	0.9	-0.052	>0.99
50 ng CCL2 vs. saline	0.56	0.02	1.01	0.074	0.075	>0.99	0.76	0.007	-0.169	0.034	-0.23	0.0002
50 ng CCL2 vs. 10 ng CCL2	0.47	0.08	1.36	0.04	0.58	0.37	0.72	0.014	-0.097	0.56	-0.2	0.0082

Table S3. Summary statistical comparison in the IM injection for myosin heavy and light chain expression. Standardized mean difference (SMD) is reported. Statistical analysis was determined by One Way ANOVA with Bonferroni post-hoc analysis. Post-hoc p-values are shown.

	MYH		MYL		MYH4	
	SMD	p	SMD	p	SMD	p
10 ng CCL2 vs. saline	-0.29	0.56	0.64	0.02	0.35	0.53
50 ng CCL2 vs. saline	-0.49	0.12	0.41	0.8	-0.77	0.75
50 ng CCL2 vs. 10 ng CCL2	-0.27	>0.99	-0.33	0.42	-0.68	0.03

Table S4. Summary table of statistical comparisons for grip strength and body mass composition in the 4T1 model with reported standardized mean difference (SMD). Statistical analysis was determined by One Way ANOVA with Bonferroni post-hoc analysis. Post-hoc p-values are shown.

	4 paw grip strength		hindlimb grip strength		lean mass composition		fat mass composition	
	SMD	p	SMD	p	SMD	p	SMD	p
4T1.Ctrl vs. healthy	-1.35	0.011	-1.23	0.02	-0.71	0.38	-1.74	0.0003
4T1.CCL2si vs. healthy	-0.88	0.31	-0.97	0.28	0.41	0.68	-0.88	0.08
4T1.CCL2si vs. 4T1.Ctrl	0.56	0.49	0.46	0.74	1.04	0.031	0.69	0.38

Table S5. Summary statistical composition in the 4T1 model for biomarker expression and CSA analysis in muscle tissues. Standardized mean difference (SMD) is reported. Statistical analysis was determined by One Way ANOVA with Bonferroni post-hoc analysis. Post-hoc p-values are shown. NA: not applicable

	CCL2 serum		CCL2 tumor		CCL2 muscle		Atrogin-1		MurF1		LC3B		CSA		Feret Diameter	
	SMD	p	SMD	p	SMD	p	SMD	p	SMD	p	SMD	p	SMD	p	SMD	p
**4T1.Ctrl vs. healthy**	1.01	0.1199	NA	NA	2.2	0.0006	1.59	0.001	2.00	<0.0001	1.34	0.21	-0.28	0.001	-0.57	p<0.0001
**4T1.CCL2si vs. healthy**	0.48	>0.9999	NA	NA	-0.11	>0.99	-0.91	0.088	-0.2	>0.9999	-0.55	>0.99	0.048	>0.99	-0.41	p<0.0001
**4T1.CCL2si vs. 4T1.Ctrl**	-0.86	0.0977	-2.52	p<0.0001	-2.24	0.0004	-3.06	<0.0001	-2.04	<0.0001	-1.91	0.046	0.29	<0.0001	0.17	0.03

Table S6. Summary statistical comparison in the 4T1 model for myosin heavy and light chain expression. Standardized mean difference (SMD) **is reported.** Standardized mean difference (SMD) is reported. Statistical analysis was determined by One Way ANOVA with Bonferroni post-hoc analysis. Post-hoc p-values are shown

	MYH		MYL		MYH4	
	SMD	p	SMD	p	SMD	p
**4T1.Ctrl vs. healthy**	-0.56	0.1	-0.27	0.55	0.242	>0.99
**4T1.CCL2si vs. healthy**	-1.06	0.001	0.29	0.42	0.12	>0.99
**4T1.CCL2si vs. 4T1.Ctrl**	-0.91	0.09	0.56	0.0081	-0.11	>0.99

Table S7. Summary statistical comparison in the 4T1 model of immune populations in muscle tissue. Standardized mean difference (SMD) and p-values are reported.

	macrophages		neutrophils		T cells		M1/CD11c+		M1/CD80+		M2/CD206+		N1/CD11c+		N1/CD80+		N2/CD206+		T helper		T regs		CTL	
	SMD	p	SMD	p	SMD	p	SMD	p	SMD	p	SMD	p	SMD	p	SMD	p	SMD	p	SMD	p	SMD	p	SMD	p
**healthy vs. 4T1.Ctrl**	-0.49	0.53	0.4	>0.99	-0.16	>0.99	0.24	>0.99	-0.73	0.86	-0.57	0.61	0.03	>0.99	-0.51	0.81	-0.41	>0.99	1.55	0.30	0.30	>0.99	-0.65	0.50
**healthy vs 4T1.CCL2si**	-0.036	0.99	1.07	0.03	0.12	>0.99	-0.79	0.50	-0.65	>0.99	-0.20	>0.99	0.35	>0.99	0.08	>0.99	0.23	>0.99	1.44	0.00	0.41	0.94	-0.15	>0.99
**4T1.Ctrl vs. 4T1.CCL2si**	0.47	0.6	0.82	0.16	0.32	>0.99	-0.49	>0.99	1.18	>0.99	0.40	>0.99	0.54	>0.99	0.71	0.60	0.67	0.49	0.78	0.12	0.17	>0.99	0.49	0.95

Table S8. Antibodies used for immunophenotyping of skeletal muscle tissue

antibody	Company, catalogue number
CD45-PE/FIRE 810	Biolegend, 157215
CD3-Alexa 700	Biolegend, 100215
F480-Pacific Blue	Bioelegend, 123123
CD11b-Spark YG-593	Biolegend, 101281
CD80-Alexa-647	Bioelegend, 104717
CD206-BV421	Bioelegend, 41717
Ly6C-BV510	Biolegend, .128033
Ly6G-APC-CY7	Tonbo Bio, 25-1276-u025
CD11c-Spark blue 550	Biolegend, 117365
CD4-FITC	Bioelegend, 100405
CD8a-APC	Bioelegend, 100711
CD25-PerCP	Biolegend, 102027

Table S9. Primary antibodies used for immunohistochemistry

antibody	Company, catalogue number
Atrogin-1	Proteintech, 67172-1-Ig
CCL2	Santa Cruz Biotechnology, 1784
LC3B	Novus Biologicals, NB100-2220SS
MuRF1	.Santa Cruz Biotechnology, sc-398608
MYH	Santa Cruz Biotechnology, 376157
MYH2	Santa Cruz Biotechnology, sc-53095
MYH4	University of Iowa Developmental Hybridoma Bank, 10F5
MYL	Santa Cruz Biotechnology, sc-365243

Disease Models & Mechanisms • Supplementary information

Table S10. Primary antibodies used for immunofluorescence and immunoblotting. *used in immunofluorescence, **used in immunoblotting

antibody	Company, catalogue number
β-actin	Sigma-Aldrich, A5441
phospho-AMPK–α	Cell Signaling Technology,2535S
AMPK-α	Cell Signaling Technology, 5832S
Atrogin1	Bioss Antibodies, 2591R
cleaved caspase-3	Cell Signaling Technology, 9661
Phospho-FOXO3 (ser253)	Thermo Fisher Scientific, PA5-118528
FOXO3*	Cell Signaling Technology, 2497
FOXO3**	ThermoFisher Scientific,PA5-118528
phospho-JNK/SAPK (Thr183/Tyr185)	Cell Signaling Technology, 8206S
JNK/SAPK	Cell Signaling Technology, 9252S
LC3B	Sino Biologicals, 14555-T52-50
phospho-p42/44MAPK	Cell Signaling Technology, 9101S
p42/44MAPK	Cell Signaling Technology, 9102S
MuRF-1	Bioss Antibodies, bs-2539R
PCNA	BioLegend, 307902
PGC-α	Santa Cruz Biotechnology, sc-518025
PPAR-γ	Santa Cruz Biotechnology, sc-7273
phospho-SMAD3 (ser423/425)	Cell Signaling Technology, 9520
SMAD3*	ThermoScientific, 66516-1-IG
SMAD3**	Cell Signaling Technology, 9523

Disease Models & Mechanisms • Supplementary information
